# Bioaminergic Responses in an In Vitro System Studying Human Gut Microbiota–Kiwifruit Interactions

**DOI:** 10.3390/microorganisms8101582

**Published:** 2020-10-14

**Authors:** Shanthi G. Parkar, Carel M. H. Jobsis, Tania M. Trower, Janine M. Cooney, Duncan I. Hedderley, Kerry L. Bentley-Hewitt

**Affiliations:** 1 The New Zealand Institute for Plant and Food Research Limited (Plant & Food Research), Private Bag 11600, Palmerston North 4442, New Zealand; carel.jobsis@plantandfood.co.nz (C.M.H.J.); duncan.hedderley@plantandfood.co.nz (D.I.H.); 2 Plant & Food Research, Private Bag 11600, Hamilton 3240, New Zealand; tania.trower@plantandfood.co.nz (T.M.T.); janine.cooney@plantandfood.co.nz (J.M.C.)

**Keywords:** green-fleshed kiwifruit, Zespri^®^ Green kiwifruit, gold-fleshed kiwifruit, Gold3 kiwifruit, Zespri^®^ SunGold kiwifruit, biogenic amines (BAs), short-chain fatty acids, SCFAs, gamma-amino butyric acid (GABA), clock gene, Brain and Muscle ARNT-Like 1 (*Bmal1*)

## Abstract

Whole kiwifruit (‘Hayward’ and ‘Zesy002’) were examined for their bioaminergic potential after being subjected to in vitro gastrointestinal digestion and colonic fermentation. Controls included the prebiotic inulin and water, a carbohydrate-free vehicle. The dopamine precursor l-dihydroxyphenylalanine (L-DOPA) and the serotonin precursor 5-hydroxytryptophan were increased in the kiwifruit gastrointestinal digesta (‘Hayward’ > ‘Zesy002’) in comparison to the water digesta. Fermentation of the digesta with human fecal bacteria for 18 h modulated the concentrations of bioamine metabolites. The most notable were the significant increases in L-DOPA (‘Zesy002’ > ‘Hayward’) and γ-aminobutyric acid (GABA) (‘Hayward’ > ‘Zesy002’). Kiwifruit increased *Bifidobacterium* spp. and Veillonellaceae (correlating with L-DOPA increase), and *Lachnospira* spp. (correlating with GABA). The digesta and fermenta were incubated with Caco-2 cells for 3 h followed by gene expression analysis. Effects were seen on genes related to serotonin synthesis/re-uptake/conversion to melatonin, gut tight junction, inflammation and circadian rhythm with different digesta and fermenta from the four treatments. These indicate potential effects of the substrates and the microbially generated organic acid and bioamine metabolites on intestinal functions that have physiological relevance. Further studies are required to confirm the potential bioaminergic effects of gut microbiota–kiwifruit interactions.

## 1. Introduction

Changes in the numbers and proportions in the trillion-plus members of the gut bacterial community are associated with biochemical, molecular and physiological changes in the host. Gut bacteria generate an array of small-molecule metabolites that, by receptor-mediated mechanisms, alter host biology. Examples of such bacterially derived ligand molecules include biogenic amines (BAs) and organic acids (OAs), the latter chiefly short-chain fatty acids [1]. Studies mainly in animal models have shown that these bacterially derived molecules send signals via the gut–brain axis to mediate effects on gut motility, visceral pain, depression, anxiety and sleep [2,3,4,5].

OAs are generated by the microbial breakdown of endogenous or diet-derived glycans and exert epigenetic control on host biology by binding to free fatty acid receptors 2/3 [6,7]. These epigenetic effects include modulation of gut wall integrity, appetite control, innate immunity, systemic lipid and glucose homeostasis [6]. OAs may also have effects on the enteric and central nervous system (ENS and CNS, respectively) owing to their ability to modulate the intestinal and systemic pools of BAs, some of which cross the blood–brain barrier [1,8,9].

The amino acids tryptophan (TRP), phenylalanine (PHE), tyrosine (TYR) and glutamic acid (GLU) serve as substrates for the generation of BA neurotransmitters. The neuronal, systemic and intestinal pools of these neurotransmitters are controlled by a complex network of genes in the host [10,11,12]. The bioaminergic pathways influence a wide range of physiological behaviors exhibiting circadian rhythmicity (e.g., sleep), and therefore show cohesive interdependencies with the canonical clock genes that control circadian-driven functions in every cell [13,14,15].

The gut microbial symbionts of the hosts are increasingly implicated in regulating BA neurotransmitter concentrations, e.g., in the PHE/TYR metabolism pathway, gut bacteria have been implicated in the generation of the CNS-regulating dopamine (DA) and the peripheral “fight or flight” hormone norepinephrine (NE) [4]. Dietary TRP is utilized by the gut bacteria to generate indole metabolites, which along with OAs signal the enterochromaffin cells to synthesize serotonin (5-HT) [8,16]. Intestinal and/or circulating catecholamines, 5-HT and indole microbial metabolites have functional roles in gut motility, nutrient absorption, immune surveillance and even glucose homeostasis [8,16,17,18]. They also play a role in influencing microbial colonization, owing to their role as quorum-sensing molecules [1,19]. Gut bacteria have also been implicated in the glutamic acid, γ-amino butyric acid (GABA) cycle, wherein glutamic acid serves as a major energy source for enterocytes or is converted to GABA [18]. GABA in turn may be microbially converted back to glutamic acid or cross the gut barrier to increase systemic pools or potentiate sleep or anxiolytic effects through the gut–vagal–brain pathways [20]. Luminal glutamic acid and GABA may also exert excitatory/inhibitory effects respectively on the CNS via vagus-mediated ligand–receptor interactions [21]. Of note also is the role of GABA as an enteric immunomodulator owing to its ability to regulate T cells and potentiate anti-inflammatory pathways [22,23]. GABA also has a key role in driving gut peristalsis [18]. GABA and 5-HT may potentially also affect the tight junctions, both in the gut wall and blood–brain barrier [24]. Another important BA is histamine, generated by the decarboxylation of histidine and other amino acids, and its role in allergy, hypertension, and immune health is well documented [1]. Other microbially generated amines such as putrescine, cadaverine, spermine and spermidine, however, have neurotoxic activity [1].

Circulating and intestinal BAs may reach concentrations between 10 µM and 1 mM, depending on physiological status and dietary intervention [25,26]. Given that BAs in this study have molecular weights ranging from 103.12 g/mol (GABA) to 220.3 g/mol (5-hydroxytryptophan (5-HTP)), circulating and intestinal BAs could range from1.0 to 2.2 µg/mL at 10 µM concentrations.

Many foods such as fruits, vegetables, nuts, meat, fish and dairy products are rich sources of BAs or their protein precursors [27]. While the BAs and most proteins are absorbed in the small intestine, some amount of protein does reach the colon and act as substrate for bacterial transformation into physiologically relevant nitrogenous compounds such as nitric oxide, BAs, phenols and ammonia [1,4,28]. Even with a very low-protein diet, 3 g of protein reaches the colon, with the major contribution being from mucosal proteins and pancreatic enzymes. With a high-protein diet, the colonic protein content may reach 18 g/day, increasing the material available for microbial biotransformation [28].

A diet rich in plant foods rapidly increases microbial diversity, and enhances families of bacteria that generate beneficial OA and BA metabolites [29]. We have previously demonstrated that green and gold kiwifruit enhance bifidobacterial populations, and drive changes in the microbial consortium to generate a cocktail of beneficial OAs. The fiber and the polyphenol components of kiwifruit were associated with this probiotic paradigm in a previously studied in vitro gastrointestinal model [30]. Kiwifruit are also sources of BA such as GABA [31], 5-HT [27] and BA precursors such as GLU, PHE, TYR and TRP [32]. Consumption of two kiwifruit one hour before sleep has been shown to improve sleep quality and duration, and this was attributed to the high content of antioxidants and 5-HT in kiwifruit [33]. Green and gold kiwifruit peel flavonoids were also found to potentiate sleep via GABA-mediated pathways [31]. Furthermore, besides these bioaminergic interactions, both green and gold kiwifruit have been shown to modulate gut microbiota from the Firmicutes phylum, many species of which have been linked with the generation of bioamines [3,25,30].

In this study, we hypothesize that metabolism of digested kiwifruit by gut microbiota will modulate the concentration of BA neurotransmitters or modulate BA-related genes in the intestinal epithelium. To address this hypothesis, we examined the changes in human fecal microbiota and microbial metabolites (BAs and OAs) generated following simulated gastrointestinal digestion and colonic fermentation of kiwifruit. The effect of kiwifruit fermenta on the expression of genes involved in BA synthesis and function was also examined using a human intestinal epithelium cell line.

## 2. Materials and Methods

### 2.1. Test Substrates

Fresh kiwifruit of the cultivars ‘Hayward’ and ‘Zesy002’ were received at the ‘ready to eat’ stage of ripeness from Zespri International Ltd., Mt Maunganui, New Zealand. *Actinidia chinensis* var. *deliciosa* ‘Hayward’ is green fleshed, marketed as Zespri^®^ Green Kiwifruit, while the gold-fleshed ‘Zesy002’ is an *A. chinensis* var. *chinensis* cultivar, marketed as Zespri^®^ SunGold Kiwifruit. The fruit were stored under refrigeration and processed within two days. They were washed with tap water, dried with a paper towel, and the proximal and distal ends of the fruit were trimmed to remove scar/wooden tissue and include only fruit tissue including skin.

The positive control was Orafti^®^ GR (hereafter referred to as inulin), a granulated powder containing a mixture of oligofructans with a degree of polymerization from 2 to 60, average ≥10 (BENEO-Orafti S.A., Oreye, Belgium). The negative control used was sterile distilled water.

### 2.2. Simulated Gastrointestinal Digestion and Colonic Fermentation

**Figure 1:**
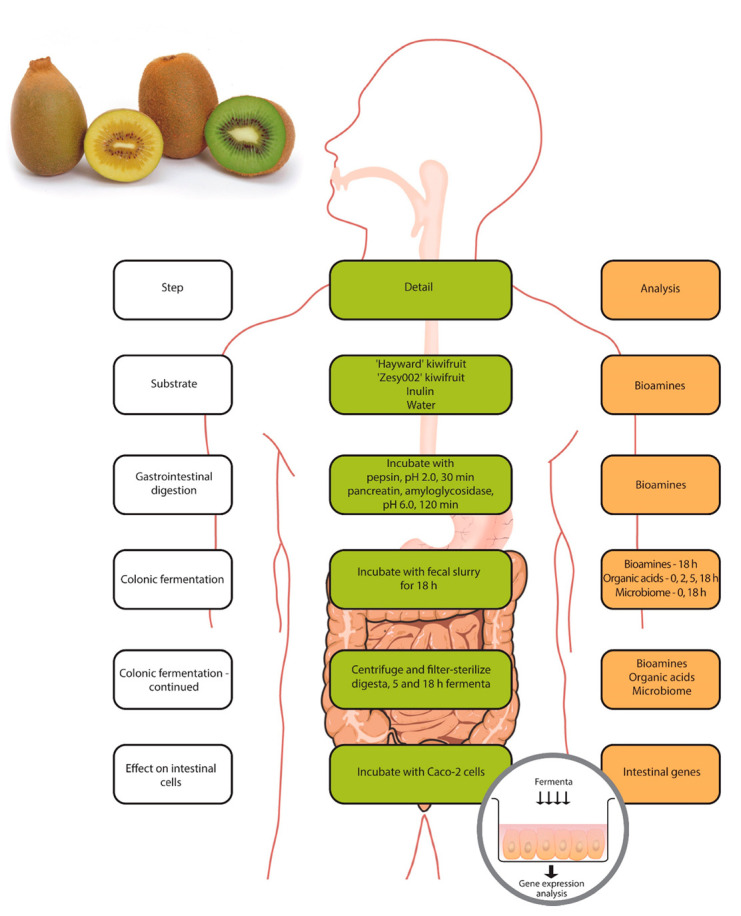
An outline of the experimental protocol used in this study.

The overall experimental protocol is as outlined in Figure 1. The fruit were sliced and coarsely blended to a puree. Then 50 g puree aliquots were subjected to a gastrointestinal digestion and fermentation protocol, as previously described [30]. Inulin and water were included as controls. Briefly, the digestion included 30 min of gastric phase (pepsin, pH 2.0) and 120 min of small intestinal phase (pancreatin, amyloglucosidase, pH 6.0) to make a final volume of 100 mL. The digesta were dialyzed with an 8–10 kDa cut-off membrane against chilled distilled water (hourly changes for the first 4 h, then left overnight with stirring).

The feces, required for the fermentation, were obtained from four healthy donors who had not taken antibiotics for the last 3 months, with approval from the Central Health and Disabilities Ethics Committee, New Zealand (13/CEN/144, approved on 11 October 2013). Samples were processed within 30 min of voiding, and blended in chilled pre-reduced glycerol-buffered saline (10 mM phosphate, 150 mM NaCl, 10% *v/v* glycerol, 0.05% *w/v* cysteine, pH 7.2) to 20% fecal slurry stocks and stored at −80 °C, as described previously [30,34].

On the day of the fermentation, one fecal slurry aliquot from each donor was thawed in the anaerobic chamber (Coy Laboratory Products Inc., Grass Lake, MI, USA), and the aliquots were mixed in equal proportions to generate a pooled fecal slurry that was immediately used for the inoculation. Then, the dialyzed digesta were added to a sterile carbohydrate-free medium, pH 6.8 and inoculated with pooled human feces at a final concentration of 1% *w/v*. The fermentation was performed anaerobically at 37 °C with shaking at 70 rpm for 18 h. The final concentration was 25 g/100 mL for the kiwifruit substrates, and 1 g/100 mL for inulin. Samples, collected as 4 × 1 mL aliquots, included the kiwifruit puree, the digesta post-gastrointestinal digestion and post-dialysis, and the fermenta at 5 and 18 h. A sample collected just before fecal slurry was added was designated as 0 h fermenta, while the fecal inoculum was separately collected. Of the aliquots, 2 × 1 mL samples were left unprocessed at −80 °C. For two aliquots, the samples were centrifuged at 16,000× *g*, at 4 °C for 5 min, and the supernatant and pellet stored separately at −80 °C.

### 2.3. Biogenic Amine (BA) Analysis

A wide range of BAs were analyzed including phenylethylamine (PEA) and its metabolites TYR, l-dihydroxyphenylalanine (l-DOPA), dopamine (DA), 3-methoxy-p-tyramine (3MT), 3,4, homovanillic acid (HVA), norepinephrine (NE), epinephrine (E), DL-metanephrine (MN), normetanephrine (NM), 3,4-dihydroxyphenylglycol (DHPG), 3-methoxy-4-hydroxyphenylglycol (MHPG), and DL-4-hydroxy-3-methoxymandelic acid (VMA). We also analyzed the TRP pathway metabolites, 5-hydroxytryptophan (5-HTP) and 5-hydroxyindole-3-acetic acid (5-HIAA) and the glutamic acid metabolite, γ-aminobutyric acid (GABA) (Figure S1).

The samples (100 µL of fruit puree, digesta and fermenta) were treated to remove proteins and derivatized in two stages to acetylate alcohol and amine functional groups and esterify acid groups, and 5 µL of the sample was then used for liquid chromatography-mass spectrometry (LCMS) analysis using an MS-probe and stable isotope coding method (detailed in supplementary methods, Figures S2–S4, Table S1).

### 2.4. Organic Acid (OA) Analysis

Organic acids (OAs, 14 linear and branched, C1 through to C7) were measured using an MS-probe and stable isotope coding LCMS method [35] with modifications [36], as detailed in supplementary methods and Table S2.

To each fermenta sample (10 µL) in an individual well of a 2 mL 96-deepwell plate (Phenomenex, Torrance, CA, USA) was added 12C/13C6-3-nitrophenylhydrazine (20 µL; 200 mM in 75:25 methanol/water *v/v*), EDC-6% pyridine (20 µL; 120 mM in methanol) and 75:25 methanol/water *v/v* (10 µL). Standards (20 µL) were treated similarly, but without the addition of the 75:25 methanol/water *v/v* (10 µL). For higher-level standards (2–40 mM), the volume of the derivatizing solutions was increased to 40 µL. Matrix spikes were prepared at physiologically relevant concentrations on each plate. The plate was sealed with a silicone mat (Phenomenex, Torrance, CA, USA) and reacted at room temperature with agitation using a ThermoMixer^®^ C (Eppendorf, Hamburg, Germany) at 1000 rpm for 45 min. The reaction was quenched by the addition of quinic acid (20 µL; 200 mM in 75:25 methanol/water *v/v*), and samples, standards and matrix spikes diluted with 10% aqueous methanol to give a total volume of 1 mL. A 100 µL aliquot of each sample, standard and matrix spike was mixed with 100 µL of the internal standard mix in a new 96-well plate, and an aliquot (10 µL) was injected for LCMS, as described in the supplementary methods.

### 2.5. DNA Extraction, Microbiome Characterization and Bioinformatics

The inoculum, i.e., the pooled fecal slurry, and the end point of the fermentation, i.e., 18 h fermenta, were used for the extraction of the DNA, which was then subjected to sequencing of the V3–V4 regions of the 16S rRNA gene using an Illumina MiSeq 2 × 250 base paired-end run (detailed in supplementary methods) [37].

The sequencing data were analyzed using Quantitative Insights Into Microbial Ecology 2 (QIIME 2, v. 2019.7) [38] in a pipeline including DADA2-based inference of sequences [39], phylogenetic placement [40], and taxonomic assignment using Greengenes database (v. 13.8, with 99% sequence similarity) [41]. Diversity analysis was performed without rarefaction, using the phyloseq package within R software, v. 3.5.0.

### 2.6. Effect of Kiwifruit on Caco-2 Cells

Human colonic adenocarcinoma cells (Caco-2, HTB-37™) were grown and the effect of the digesta and fermenta on the cell viability tested as detailed in the supplementary methods.

The assay investigating the effect of the kiwifruit on Caco-2 gene expression is detailed in supplementary methods. Briefly, on the day of the assay, the Caco-2 cells were synchronized to the same circadian phase by pre-incubation with 50% fetal bovine serum in cell culture media for 2 h [42], and then washing with buffered saline. Cells were then incubated for 3 h with filter-sterile 20% dilutions of 0, 5 and 18 h fermenta or pooled fecal inoculum. The 0 h fermenta which did not contain fecal inoculum served to examine the effect of the digested substrates. The fecal inoculum was included as a control to test the effect of the fecal bacteria. Caco-2 cells incubated only with background cell culture medium were also included as controls. The washed cell lysates were used for extraction of RNA for gene expression studies.

RNA samples (194 ng) were analyzed using the nCounter^®^ Plexset™ reagents (NanoString, Seattle, WA, USA) following the manufacturer’s instructions. Target sequences were designed by NanoString Technologies, Inc. and ordered from Integrated DNA Technologies, Inc., Iowa, USA (Table S3). Samples were spiked with six different internal control probes at concentrations ranging from 128 to 0.125 fM in four-fold dilution steps. Eight positive controls were used to determine the linearity of the assays and were used for normalization. Eight negative probes were used to control for carryover of reporter probes as no RNA target was included for these probes. RNA samples were incubated for 24 h at 65 °C in hybridization buffer containing the CodeSet, which consisted of reporter and capture probes and together with the target RNA formed a tripartite complex. After hybridization, the complex was bound by its biotin-labelled capture probe on a streptavidin-coated glass slide and was stretched within an electric field. Hybridized samples were processed using the robotic Prep Station (High Sensitivity Protocol, 3 h per 12-sample cartridge) and data acquisition was performed by using the GEN2 Digital Analyzer (nCounter^®^, Washington, DC, USA), with the ‘Max’ Field of View setting (555 images per sample; 5 h scan per cartridge). Raw counts were normalized using the positive controls, and target genes were normalized to the five internal reference genes (Table S3).

### 2.7. Statistical Analysis

For analysis of BAs and the OAs, gamma-distribution generalized linear models (GLM, using stats package in R) were fitted to examine changes in terms of the stage of digestion (undigested substrate, gastric, intestinal and dialyzed digesta, and fermenta) followed by testing the difference between the various substrates at the stage of fermentation. Where there were significant differences (*p* < 0.005) in either step, Tukey’s honest significant test (*p* = 0.05) was performed. For the microbiome data, 18 h fermenta DNA were analyzed to examine differences of each substrate with respect to the water control using DESeq2 [43], as detailed in supplementary methods. RNA data were analyzed using DESeq2 [43] with likelihood ratio tests and a nested factorial structure which allowed testing for differences between the treatments at each time point, and then between the averages for each time point. The *p* values adjusted for false discovery rate are quoted. Spearman correlations were calculated to examine relationships between OA and BA concentrations, and BA and microbiome using stats package in R.

## 3. Results

### 3.1. Biogenic Amines (BAs)

**Figure 2:**
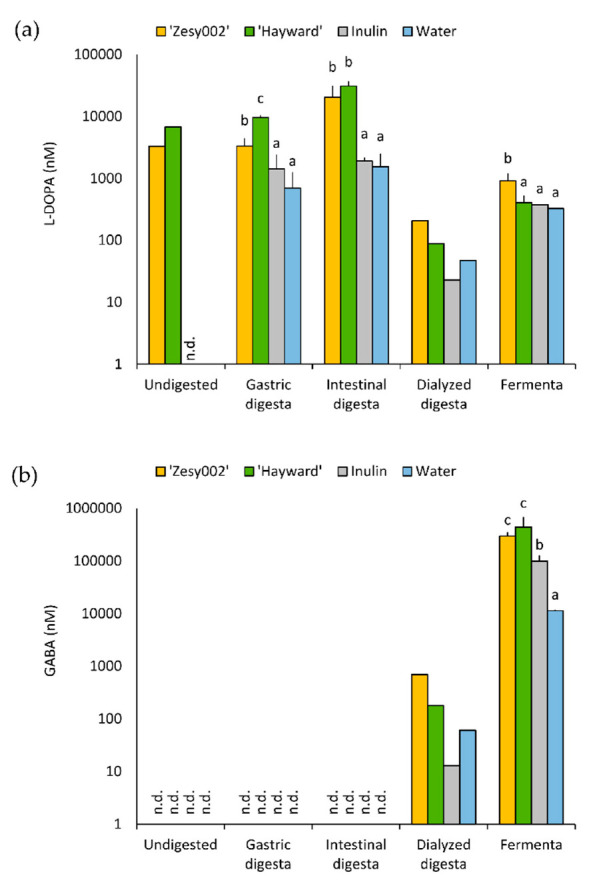
Concentrations of (**a**) l-dihydroxyphenylalanine (l-DOPA) and (**b**) γ-amino butyric acid (GABA) (**b**) after various stages of gastrointestinal digestion and fermentation. Values are the means ± standard deviation. In case of the undigested samples, only ‘Hayward’, ‘Zesy002’ and water samples were analyzed, and only singly. The dialyzed digesta was also measured singly. There were three samples (*n* = 3) for gastric and intestinal digesta and fermenta. When there were significant changes between substrates (*p* < 0.005, GLM) at a stage of digestion or fermentation, Tukey’s test was performed. Within a type of digesta, means with letters in common are not significantly different (Tukey’s test, *p* = 0.05). n.d., not detectable.

Of the sixteen BAs studied, there were significant effects on the concentration of a number of these compounds during different stages of digestion and colonic fermentation depending on the substrate (Figure 2 and Figure S5). BAs that achieved concentrations greater than 10 µM in at least one stage of the gastrointestinal model, with significant treatment effects (*p* < 0.005, GLM) included TYR (gastric and intestinal stages), l-DOPA (at every gastrointestinal stage), and GABA (at the 18 h fermenta stage). In the TYR metabolic pathway, TYR increased in the ‘Hayward’ treatment, with gastric and intestinal digesta concentrations being five-fold and four-fold those of the water control values (*p* < 0.05, Tukey test) (Figure S5). l-DOPA, which is generated from TYR, was increased in ‘Zesy002’, with 5-, 13- and 3-fold increases in the gastric, intestinal and fermenta stages, respectively, in comparison to the respective water control values (*p* < 0.05). ‘Hayward’ showed 14- and 20-fold increases compared with the water control values at the gastric and intestinal stages, respectively, but with no change after fermentation. (Figure 2a). GABA was increased in ‘Hayward’ fermenta at 39 times the water fermenta values. ‘Zesy002’ and the inulin control also increased GABA to approximately 26 and 9 times the water control values (*p* < 0.05) (Figure 2b).

### 3.2. Organic Acids (OAs)

Analysis of OA concentrations revealed that there were significant changes (*p* < 0.005, GLM) driven by the nature of the substrates and the fermentation time (i.e., 2, 5 and 18 h) (Table 1 and Figure S6). At the end of 18 h in comparison to the water control, both lactic and acetic acids were increased by ‘Hayward’ and ‘Zesy002’ over inulin and water (*p* < 0.05, Tukey). Propionic and butyric acid were increased most by ‘Hayward’ and inulin respectively (Table 1). The water fermenta was highest in valeric, isobutyric, 2-methyl butyric and 3-methyl valeric acid (Table 1). Spearman correlation analysis (*r* ≥ 0.7, *p* = 0.01) between the three major BAs and the four OAs revealed that lactic acid correlated with TYR and GABA, while acetic acid correlated with GABA. Butyric acid negatively correlated with TYR.

### 3.3. Microbiome

**Figure 3:**
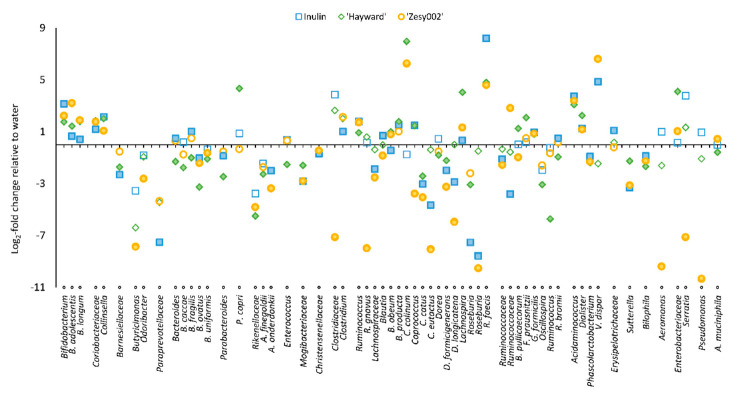
Changes in microbial abundances in the 18 h fermenta of ‘Hayward’, ‘Zesy002’ and inulin at the genus/family level in comparison with the water control. The filled data points indicate significance (*p* < 0.001) in comparison to the water control.

Microbiome analysis was performed for 18 h fermenta and the fecal inoculum. Diversity analysis within samples (α-diversity) and between samples (β-diversity) revealed that time (inoculum versus 18 h of fermentation) and substrate (after 18 h of fermentation) were significant influencers (supplementary results; Figures S7–S11). Differential abundance analysis using DESeq2 was performed, and the values for the 18 h fermented carbohydrate-rich substrates, ‘Hayward’, ‘Zesy002’ and inulin were expressed as relative changes in comparison to the water control (Figure 3 and supplementary Table S4). All three substrates increased bacteria, including *Faecalibacterium prausnitzii*, *Roseburia faecis*, *Bifidobacterium* spp., *Clostridium* spp., *Ruminococcus* spp., *Acidaminococcus* spp., and *Lachnospira* spp., and decreased *Clostridium eutactus*, *Butyricimonas* spp., *Odoribacter* spp., *Alistipes* spp., *Ruminococcus* spp., *Roseburia* spp., *Oscillospira* spp., and unclassified genera of Paraprevotelloaceae, Rikenallaceae, Barnesiellaceae and Lachnospiraceae. Other observations were that ‘Hayward’ induced increases in *Prevotella copri*, *Veillonella* spp., and decreases in *Bacteroides* and unclassified Rikenellaceae. ‘Zesy002’ induced increases in *Ruminococcus bromii*, *Bacteroides* and Bifidobacteriaceae and decreases in *Bacteroides ovatus*, *Alistipes onderdonkii* and unclassified Rikenellaceae. Inulin induced increases in *Bacteroides fragilis*, *Ruminococcus bromii* and unclassified *Bacteroides* and a decrease in *Bacteroides ovatus*.

Spearman correlational analysis was used to study correlations between BAs and gut bacteria (*r* ≥ 0.73, *p* = 0.01; **bold** font is used when *r* ≥ 0.85, *p* = 0.001). l-DOPA correlated positively (*r* ≥ 0.73) with *Bifidobacterium* spp. and two Veillonellaceae, i.e., *Veillonella dispar* and *Acidaminococcus* spp. and negatively with ***Ruminococcus gnavus***, *Coprococcus eutactus*, *Dorea longicatena* and *Roseburia* spp. Significant positive correlations were seen between GABA and *Lachnospira* spp. and negative correlations were seen with unclassified species of *Blautia*, *Coprococcus*, *Butyricimonas* and *Anaerotruncus*. Examining the microbiome changes in this context, *Acidaminoccus* increased by multiple log_2_fold changes (FC), compared with the water control in the presence of ‘Hayward’ (3.1), ‘Zesy002’ (3.1) and inulin (3.7). *Bifidobacterium* spp. increased with ‘Hayward’ (1.8 log*2* FC), ‘Zesy002’ (2.2) and inulin (3.2). Presence of ‘Zesy002’ and inulin also increased *V. dispar* (6.6 and 4.9 log*_2_*FC respectively), ‘Zesy002’ fermenta was 2-fold higher in l-DOPA than those of ‘Hayward’ and inulin. *Lachnospira* spp. increased most with ‘Hayward’ (4.0 log_2_FC) compared with ‘Zesy002’ (1.3) and inulin (0.3), and indeed the GABA concentrations were higher in ‘Hayward’ (4) and ‘Zesy002’ (3) fermenta than in inulin fermenta. *Butyricimonas* was suppressed most in ‘Zesy002’ and ‘Hayward’ (5.0) fermenta, followed by inulin (3.5). *Coprococcus* spp. was suppressed in ‘Zesy002’ fermenta but increased in ‘Hayward’ and inulin fermenta. The effect of the substrates on *Blautia* spp. were within a 0.8 log_2_FC, and there was no significant effect on *Anaerotruncus* by any of the substrates.

### 3.4. Intestinal Gene Expression

**Figure 4:**
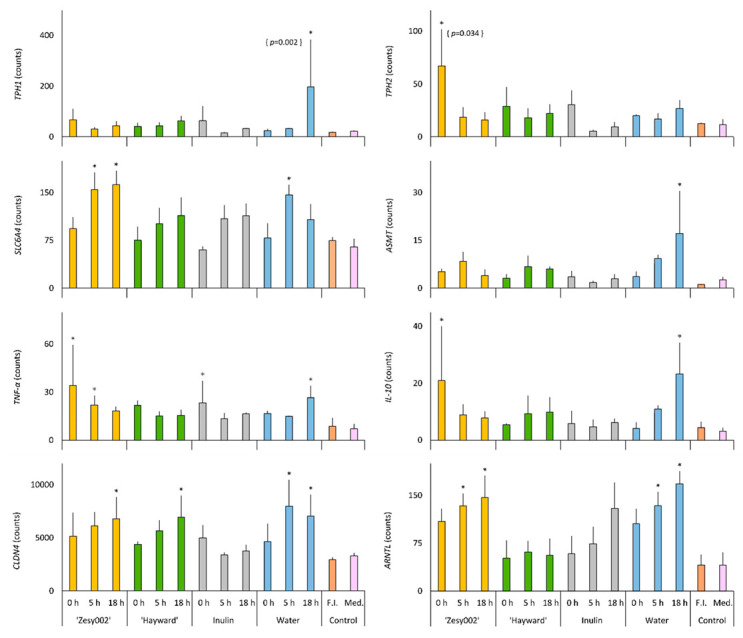
Gene expression data from Caco-2 cells incubated for 3 h (*n* = 3) with kiwifruit, inulin or water fermenta collected at 0 h (minus fecal inoculum), 5 and 18 h, or the controls, i.e., fecal inoculum (F.I.) used for the fermentation experiment, and the background medium (Med.). The genes studied include the tryptophan hydroxylase isoforms, *TPH1* and *TPH2*, serotonin transporter *SLC6A4*, *N*-acetylserotonin *O*-methyltransferase *ASMT*, tumor necrosis factor-α *TNF-α*, interleukin-10 *IL-10*, claudin4 *CLDN4* and clock gene *ARNTL1*. Values are the mean counts ± standard errors of means. The counts have been normalized to five reference genes. Significant difference (*p* < 0.05) from medium control is indicated by *. Significant differences between fermenta within a substrate group were *p* = 0.034 for *TPH2* with ‘Zesy002’ and *p* = 0.002 for *TPH1* with water.

We examined the effect of the fermenta on the expression of intestinal genes using a confluent Caco-2 monolayer as a model of the intestinal epithelium (Figure 4, Table S5). Comparisons between fermenta within a substrate group, including all fermenta time points, showed an increase in the expression of the tryptophan hydroxylase isoforms, *TPH1* and *TPH2,* which are genes regulating the concentration of 5-HT (*p* = 0.034 for *TPH2* with ‘Zesy002’ and *p* = 0.002 for *TPH1* with water). Further comparisons of the treatments were performed with the untreated background medium control and deemed significant when *p* > 0.05. Of the genes regulating the concentration of bioamines, *TPH1* was increased by the 18 h water fermenta, *TPH2* was increased by the ‘Zesy002’ 0 h fermenta and serotonin transporter *SLC6A4* was increased by 5 h water fermenta and the 5 and 18 h ‘Zesy002’ fermenta, while the *N*-acetylserotonin *O*-methyltransferase gene (*ASMT*) was increased by 18 h water fermenta. The aryl hydrocarbon receptor nuclear translocator-like gene (*ARNTL*) was increased by the 5 and 18 h fermenta of ‘Zesy002’ and the water control, while the period2 (*PER2*) gene was decreased only by the 18 h ‘Zesy002’ fermenta (Table S5). An examination of the inflammatory status of the gut cells showed that the interleukin-10 (*IL-10*) gene was increased by the 5 h water fermenta and the 0 h ‘Zesy002’ fermenta, while the pro-inflammatory tumor necrosis factor-α (*TNF-α*) was increased by water fermenta (5- and 18 h) and ‘Zesy002’ fermenta (all three time points) and ‘Hayward’ fermenta (0 h). Of the intestinal tight junction genes, *CLDN4* was increased by water fermenta (5 and 18 h), and 18 h fermenta of both kiwifruit cultivars.

## 4. Discussion

The bioaminergic potential of whole green and gold kiwifruit cultivars is so far unreported. This is the first study to demonstrate that ‘Hayward’ and ‘Zesy002’ stimulate gut microbiota and microbial metabolites that may potentially influence the intestinal BA pool. Furthermore, we demonstrate effects of kiwifruit–microbial interactions on intestinal genes that regulate BA synthesis, or mediate bioaminergic physiological effects.

### 4.1. Kiwifruit Digesta as Source of Biogenic Amines (BAs) and BA Precursors

We anticipated that the BAs of kiwifruit origin would be retained in the gastric and intestinal digestion stages but removed by dialysis using 8–10 kDa membranes (a simulation of passive, but not active transport across the intestine). The increases in TYR and its metabolites l-DOPA, DA and the 5-HT precursor 5-HTP in ‘Hayward’ and ‘Zesy002’ digesta (i.e., unfermented) indicate that these BAs are generated by breakdown of the fruit during gastrointestinal transit and therefore, are potentially available for small intestinal absorption. For example, TYR and l-DOPA have been known to be rapidly absorbed in the small intestine, with approximately 1% of l-DOPA crossing the blood–brain barrier to be decarboxylated to DA [44].

Although the glutamic acid-derived GABA has been detected in the gold kiwifruit peel extracts [31], GABA was not detected in the digested kiwifruit, with the exception of the dialyzed digesta, which may indicate either a concentration effect or that detection was confounded owing to the presence of other compounds such as sugars or soluble fiber.

Other whole food BA sources—spinach (5-HT and GABA), banana (DA) and tomato (5-HT)—have been implicated as possible dietary means of modulating cognition, mood and sleep [27,31,33]. Kiwifruit are rich sources of precursor amino acids including TYR, TRP, PHE and GLU [32]. This study and other literature indicate that kiwifruit may also provide a source of dietary BAs that are known to have potential behavioral effects [27,32].

### 4.2. Role of Microbiota–Kiwifruit Interactions in Producing Organic Acids (OAs) and Biogenic Amines (BAs)

The kiwifruit dose for this in vitro gastrointestinal study assumed a serve size of 200 g kiwifruit (approximately two kiwifruit) and an intestinal volume of 400 mL [45], and therefore is physiologically relevant. The major contributions from ‘Hayward’ and ‘Zesy002’ to the in vitro fermentation stage would be approximately 0.8 and 0.6 g fiber/100 mL respectively (based on previous analyses [30,32]) and polyphenols such as the prebiotic polyphenolics that also escape digestion [30,32]. The kiwifruit fiber have also been shown to present a substrate of greater complexity in terms of sugar composition and linkages in comparison to the oligofructan, inulin [46]. The inulin was used at concentration of 1 g/100 mL, a dose previously estimated to be prebiotic in similar fecal fermentation protocol [30,46]. Thus, the fiber presented to the fecal bacteria may be of similar magnitude in terms of concentration, but not composition, and this may have a bearing on microbial fermentation rates and pathways.

At the end of 18 h of fermentation, the higher concentrations of early metabolites of carbohydrate fermentation, i.e., lactic and acetic acid in ‘Hayward’ and ‘Zesy002’ fermenta, indicate the availability of the fiber (‘Hayward’ > ‘Zesy002’) for microbial metabolism [30]. When the monosaccharides are depleted, the 3-carbon and 4-carbon propionic and butyric acids respectively, are formed (very clearly evident in inulin), by bacteria with the metabolic capacity to utilize the early intermediates, and break down more complex carbohydrate linkages [7]. It also explains the higher diversity of ‘Zesy002’ over ‘Hayward’, as the community in the latter utilize the still available sugars, rather than diversifying to utilize the primary metabolites. The water vehicle control, however, has a unique metabolite profile of higher concentration of branched-chain fatty acids. This is due to a lack of carbohydrates in the fermenta, with the glycoproteins from the digestive enzymes added during the digestion phase and the peptones in the media acting as energy sources for bacteria such as Bacteroidetes and Enterobacteriaceae, which are capable of proteolytic metabolism [30,47]. The increased microbial diversity in the water fermenta is a reflection of the increased co-operation in the microbial community to ensure its survival.

As the kiwifruit or other BA-rich fruit transit the gastrointestinal tract, small molecules such as sugars and BAs will be absorbed at the small intestinal stage, with the remnant fiber and unabsorbed protein reaching the large intestine. The vast and complex microbial community in the colon extract energy from the fiber and protein to generate OA and BA microbial metabolites, which in turn provide a source of energy and stimulate bioaminergic responses in colonocytes or are absorbed [2,25,48]. The systemic pool of BAs may thus be of dietary and/or gut microbial origin, with effects on the body via the ENS or systemic circulation. The increases in fermenta concentrations of l-DOPA (‘Zesy002’ > ‘Hayward’) and GABA (‘Hayward’ > ‘Zesy002’) and to a lesser extent even inulin, indicate the potential of gut microbiota to generate BAs. Some BAs, such as TYR and its metabolite, l-DOPA, and TRP and its metabolites, 5-HTP and 5-HIAA, are also known to cross the blood–brain barrier and therefore have central neurological effects, while current evidence leans more towards the effect of GABA being mediated mainly through the gut–vagal–brain axis, while not ruling out blood–brain barrier passage [12,20,24,27,43]. GABA is generated by both host- and microbially mediated decarboxylation of glutamic acid, and serves to downregulate pro-inflammatory responses, and potentiate gastrointestinal motility, gut–brain signaling through the vagal nerves and circadian rhythm [15,21,49].

The prebiotic inulin, when fed to piglets, modulated the concentrations of colonic bioamines, one of which was phenylethylamine, a precursor in the TYR pathway [26], indicating the potential connections between fiber-induced microbial biotransformation as a source of intestinal BAs. Inulin thus served as a positive control due to its prebiotic potential, to validate the in vitro fermentation and its ability to stimulate gut bacteria to generate BAs. We detected similar correlations. l-DOPA formation was associated with *Bifidobacterium* spp. (a lactate producer) and the lactate-metabolizing Veillonellaceae, *V. dispar* and *Acidaminococcus* spp. Veillonellaceae typically increase in the early steps of substrate degradation as they have limited ability to degrade carbohydrates and instead rely on early metabolites from other bacteria for their sustenance [50]. *V. dispar* is known to grow on lactate and some bioamines such as cadaverine, while acidaminococci grow solely on amino acids (mainly glutamic acid), indicating interactions with BA pathways [51]. Select species of Bifidobacteriaceae have also been implicated in the hydroxylation of TYR to l-DOPA [52], and thus the higher numbers of *Bifidobacterium* spp. in ‘Zesy002’ (vs ‘Hayward’) fermenta may contribute to its higher l-DOPA compared to ‘Hayward’. This overall increase in l-DOPA may provide an intestinal source of substrate for host/microbial transformation to DA, and even be available for decarboxylation to DA after crossing the blood–brain barrier [44].

Microbiota–GABA interactions could similarly be inferred, especially with kiwifruit which increased GABA concentrations much more than inulin. Indeed, ‘Hayward’ and ‘Zesy002’-induced increases in *Lachnospira* spp., the organism that correlated significantly with GABA production. GABA production by gut commensals has been suggested to be a stress response to counter acidic environments by decarboxylation of glutamic acid at a low pH, and has been demonstrated in lactic acid bacilli, *Bacteroides* spp., and clostridia, all of which are enriched in a microenvironment such as that generated by kiwifruit [4,30]. Gut commensals thus play an important role in regulating the luminal glutamic acid/GABA ratio, which in turn influences circadian-controlled pathways of inflammation, glucose metabolism and sleep [21].

### 4.3. Effect of Fermenta on Caco-2 Gene Expression Responses

The *TPH* gene expresses the rate-limiting enzyme for 5-HT biosynthesis, of which the *TPH1* isoform is located in the gut epithelial cells as well as the enterochromaffin cells, while *TPH2* gene is expressed only in neuronal cells of the gut and the brain [53,54]. Serotonin has many beneficial roles in gut motility and innate immunity, but in excess may mediate pro-inflammatory states by sending quorum sensing signals that enhance bacterial pathogenicity [18]. The balance between the *TPH* pathway and the competing kynurenine synthesis may thus have a bidirectional relationship with the immunological fate in the gut and even beyond via the gut–brain axis [18]. The *TPH1* increase by the carbohydrate-free water fermenta, may be at least partially be an effect of the acid milieu [47]. The fermentation of water represents a typical distal-gut metabolic state, where the carbohydrates are scarce and the microbiota resort to extraction of energy from remnant proteins, resulting in the generation of branched-chain fatty acids [47]. While there is no direct evidence of the role of branched-chain fatty acids, the colonic enterochromaffin cells that release 5-HT are highly enriched in specialized receptors for branched-chain fatty acids [55]. This potential *TPH1*-modulating role of intestinal branched-chain fatty acids is yet to be studied but indicates an opportunity for microbiota to influence bioamine pools in the distal colon. Kiwifruit fermenta (in the given dilution) did not affect *TPH1* expression, at least at the end of 3 h. The significance of the increased *TPH2* by unfermented ‘Zesy002’ is unclear. The serotonin transporter *SLC6A4* is expressed in the enterocytes to enable 5-HT re-uptake [8]. Inhibition of *TPH1* and cellular requirements for 5-HT have been suggested to upregulate the expression of *SLC6A4* as a compensatory response [8]. The increased expression following the applications of ‘Zesy002’ and water fermenta on Caco-2 cells may at least partly have been due to the suppressed *TPH1*, a cellular mechanism that has been suggested to be adapted to conserve 5-HT [8]. This is consistent with a similar, but statistically not significant, increase in *SLC6A4* with ‘Hayward’ and inulin fermenta-treated cells.

We also examined expression of *ASMT* gene, the key enzyme that catalyzes the conversion of 5-HT to melatonin in the intestinal cells, mainly the enterochromaffin cells [56]. Melatonin has potent anti-inflammatory functions, and protects the gut barrier [16]. Applications of fermenta from the prebiotic inulin and the kiwifruit did not affect *ASMT* expression, indicating no fiber-driven changes in *ASMT*. Water fermenta upregulated *ASMT*, the reason for which is not clear, but may include unique metabolic profile driven by a carbohydrate-poor protein substrate.

Bioaminergic immunomodulation of innate immunity is well recognized, and evident in the gut, and even in the CNS mediated by the gut–brain pathways [9,16,18,47]. The pro-inflammatory cytokine encoding gene *TNF-α* was upregulated by unfermented ‘Zesy002’ and inulin. The decrease in TNF-α by the fermented counterparts may be related to the post-fermentation increases in GABA (26- and 9-fold in ‘Zesy002’ and inulin respectively as compared to water), or a microbiota-mediated breakdown of pro-inflammatory components. The increased *TNF-α* in ulcerative colitis patients has been associated with decreased microbial production of GABA [22], indicating a role for gut microbiota-derived GABA in TNF-α-mediated inflammations. Furthermore, studies with mice models of colitis have showed that exogenously added GABA attenuated the colitis-induced TNF-α increase and instead increased the production of the anti-inflammatory cytokine IL-10 [23]. One more differentiating factor between the carbohydrate-fermentation (kiwifruit and inulin) and proteolytic fermentation (water control) is the proportion of acid metabolites. Butyric acid and to a lesser extent, propionic acid are potent inhibitors of pro-inflammatory histone deacetylases, and therefore push immunological events towards decreasing production of TNF-α and increasing IL-10. The water fermenta contained higher concentrations of branched-chain fatty acids that have been associated with low-grade inflammation such as that observed in obesity and diabetes [57]. The *TNF-α* increase may thus be in response to the concentrations of branched-chain fatty acids and GABA, which were the highest and the lowest respectively in the water fermenta (in comparison to the other fermenta). ‘Hayward’ digesta did not increase *TNF-α* as much as ‘Zesy002’, but with a similar trend in terms of decrease in TNF-α after fermentation was observed.

Microbiota-mediated enteric bioaminergic signaling may influence the integrity of the intestinal epithelium, compromise of which has been associated with inflammation [22,53]. The increase in the expression of *CLDN4*, which expresses the claudin4 tight junction protein, with fermenta may imply a role for microbial metabolites in modulating the gut wall physicochemical structure. The increased *CLDN4* expression seen after the application of the kiwifruit fermenta may be attributed to GABA and/or the acid metabolites. GABAergic mechanisms have been implicated in a recent study where GABA-producing probiotic was found to increase the expression of *CLDN4* (and other tight junction genes) in Caco-2 cells [58]. Thus, the high GABA in both ‘Zesy002’ and ‘Hayward’ fermenta may contribute to the upregulated *CLDN4* expression. Short-chain fatty acids, such as those microbially generated in a carbohydrate-rich milieu also contribute to the gut barrier integrity by upregulating tight junction genes (in the order of butyric ≥ propionic > acetic acid) [48]. This role of the acid metabolites may also explain the effect of the water fermenta in influencing the *CLDN4* expression.

Strong interdependencies have been observed between circadian rhythm, bioamines and behaviors such as the sleep/wake cycle and food intake, with metabolic consequences on health [3,13,14]. Circadian rhythm, regulated by a conserved set of clock genes, is entrained not just by light (the main Zeitgeber) but also by peripheral organs and the gut microbiota [2,3,15]. Mixtures of lactic, acetic, propionic and butyric acids, either when fed orally or increased in the mice ceca after a high-fiber diet, were found to induce significant phase advances of peripheral clock genes in mice [2]. Clock genes such as *ARNTL* may also show sensitivity to the bioamines that are present in these fermenta [59,60]. A*RNTL,* also called as Brain and Muscle ARNT-Like 1 (*Bmal1*) is the only non-redundant clock gene, and is essential for regulation of mammalian processes including BA synthesis [60]. The *ARNTL* expression increased only with the fermented ‘Zesy002’ and the water control implies potential microbiota-mediated effects. This may be attributed to the metabolomic profile, including the acid and amine metabolites in these fermenta, or a delay or advancement of changes that were not captured in this single time point study. Further expression studies over a 24 h period are required to confirm the microbiome-*ARNTL1* link, given the cyclical nature of expression of *ARNTL1* expression [60].

Summarizing the gene expression studies, ’Zesy002’ unfermented digesta increased Caco-2 expression of *TPH2*, *TNF-α* and *IL-10*, and these changes were ameliorated after fermentation. A similar effect was seen with inulin for *TNF-α*. ‘Hayward’ digesta did not affect any of the genes, and the fermenta only enhanced *CLDN4*. Most changes were observed with *CLDN4*, which was upregulated by 18 h fermenta of both the kiwifruit and the water. *ARNTL* was increased by ‘Zesy002’ and water fermenta. The water fermenta enhanced *TPH1*, *SLC6A4*, *TNF-α*, *IL-10*, *CLDN-4* and *ARNTL*, and some of these effects may be attributed to the concentration and proportion of the acid and amine metabolites generated during the proteolytic fermentation pathways adopted by gut bacteria in a carbohydrate-free environment.

### 4.4. Merits and Limitations of the Study

A batch fermentation model with a pooled human fecal inoculum was used in this study and does not simulate the complex processes of fermentation or metabolite absorption of the colon. However, this model is a relatively rapid method, simulating upper and lower gut processes of food digestion, to examine the prebiotic potential of whole foods [30]. The Caco-2 cell is a reductionist simulation of the intestinal epithelium. The intestine is a complex organ with heterogeneous cell types and vasal/neuronal connections to the peripheral organs and brain. However, by using filter-sterile fermenta and limiting incubations to 3 h, we were able to capture a one time-point snapshot of potential intestinal responses to microbiota–kiwifruit interactions. Whole kiwifruit was used for the study, rather than a kiwifruit extract or fraction. While kiwifruit peel is not necessarily consumed, it was included in our whole kiwifruit study, as kiwifruit peel has previously been shown to contain bioactives [31,61]. The array of neuroactive BAs analyzed did not include 5-HT, melatonin, histamine and many trace amines. A measure of serotoninergic involvement was, however, deduced through measurement of its precursor 5-HTP, and analysis of *TPH* gene (5-HT biosynthesis) and *SLC6A4* (intracellular 5-HT uptake). Further, changes in mRNA transcription are not necessarily a reflection of the post-transcriptional changes or generation of the enzyme. Further studies examining gene and the enzyme outputs would be required to confirm this study.

## 5. Conclusions

Both kiwifruit cultivars are sources of BAs even before fecal fermentation, with the most notable being DA precursor l-DOPA and the 5-HT precursor 5-HTP, with the ‘Hayward’ digesta generating more of both the named BAs than the ‘Zesy002’ digesta.

After 18 h of fecal fermentation, kiwifruit and to some extent inulin enhanced generation of BAs. ‘Zesy002’ caused a greater increase in l-DOPA while ‘Hayward’ favored GABA production, and these changes were associated mainly with increases in bifidobacteria and Firmicutes such as Veillonellaceae and Lachnospira. Kiwifruit thus drive a probiotic consortium-mediated production of BAs which may potentially influence host biology.

Upon incubation with Caco-2 cells, the kiwifruit and water fermenta upregulated *CLND4*, supporting a potential role of acid metabolites with intestinal integrity. ‘Zesy002’ digesta and fermenta increased the expression of *TPH2* and *SLC6A4*, implying ‘Zesy002’-induced changes in the serotonergic pathway. The ‘Zesy002’ and water fermenta induced increases in the expression of the canonical clock gene *ARNTL4*, indicating a potential circadian role of ‘Zesy002’–microbiota interactions. Water fermenta also affected many intestinal genes, and this may be attributed to the peculiar fermentation characteristics of a carbohydrate-free, protein-rich milieu.

It thus appears that kiwifruit substrates may enhance gut microbiota-driven acid metabolite and neurochemical profiles that are associated with immune, metabolic and circadian health. Clinical studies on the connection between kiwifruit consumption and the bioaminergic effects on the physiology are required to confirm the results from this preliminary in vitro study.

## Figures and Tables

**Table 1:** Organic acid concentrations after 18 h of fermentation.

Organic Acid	‘Zesy002’	‘Hayward’	Inulin	Water	Average CV (%)
Formic acid	4.01 ^a^	1.03 ^b^	0.93 ^b^	0.14 ^c^	34
Lactic acid	2.94 ^a^	0.78 ^b^	0.03 ^c^	0.02 ^c^	64
Acetic acid	19.04 ^a^	26.42 ^a^	6.41 ^b^	11.22 ^c^	26
Propionic acid	1.68 ^a^	6.32 ^b^	1.97 ^a^	4.45 ^c^	13
Butyric acid	3.21 ^a^	7.35 ^b^	27.22 ^c^	14.21 ^d^	23
Succinic acid	0.02 ^a^	0.00 ^b^	0.00 ^bc^	0.00 ^c^	45
Isobutyric acid	0.03 ^a^	0.04 ^a^	0.05 ^b^	0.20 ^c^	14
2-methyl butyric acid	0.02 ^a^	0.02 ^a^	0.04 ^b^	0.22 ^c^	14
Isovaleric acid	0.03 ^a^	0.02 ^a^	0.04 ^a^	0.21 ^c^	11
Valeric acid	0.02 ^a^	0.04 ^b^	0.08 ^c^	0.15 ^d^	14
2-methyl valeric acid	0.00 ^a^	0.00 ^a^	0.00 ^a^	0.01 ^b^	30
Caproic acid	0.01 ^a^	0.01 ^a^	0.01 ^b^	0.02 ^b^	7
Heptanoic acid	0.001 ^a^	0.003 ^b^	0.003 ^b^	0.004 ^b^	15

Values are the mean organic acid concentrations (mM) of fermenta after 18 h of fecal microbial fermentation (*n* = 3). The average coefficient of variability (CV) expressed as a percentage is also presented. The significance values adjusted to the false discovery rate were found to be *p* < 0.005 for all the acids. Within an organic acid, means with letters in common are not significantly different (*p* = 0.05, Tukey’s test).

## Data Availability

The 16S rRNA gene sequencing data files are available in the Sequence Read Archive held by NCBI database (BioProject ID PRJNA662182).

## Supplementary Materials

The following are available online at https://www.mdpi.com/2076-2607/8/10/1582/s1, Figure S1: Schematic outlining the biogenic amines generated by the tryptophan, tyrosine and the glutamate metabolism pathways, Figure S2: The derivatization schematic for the LC-MS method to analyze biogenic amines, Figure S3: Workflow prior to LC-MS analysis of biogenic amines, Figure S4: Transitions monitored during LC-MS quantitation of biogenic amines, Figure S5: Concentrations of different bioamines after various stages of gastrointestinal digestion and fermentation of kiwifruit, Figure S6: A principal components analysis plot visualizing the influences of the substrates in generating organic acid metabolites over the duration of the fecal microbial fermentation (0, 2, 5 and 18 h), Figure S7: Mean relative frequency of microbiota of samples at phylum level, Figure S8: Mean relative frequency of microbiota of samples at order level, Figure S9: Mean relative frequency of microbiota of samples at family level, Figure S10: Diversity analysis examining changes in microbial community within samples, Figure S11: Principal co-ordinate analysis plot demonstrating β-diversity differences between samples, based on the Bray-Curtis index, Table S1: Multiple Reaction Monitoring (MRM) transitions used for biogenic amine analysis, Table S2: Multiple Reaction Monitoring transitions used for organic acid analysis, Table S3: Intestinal gene targets analyzed, Table S4: Relative changes in microbiome abundance in fermenta after 18 h of fermenta, Table S5: Mean counts of Caco-2 cell genes.
