# Supplementary material for: Bioaminergic Responses in an In Vitro System Studying Human Gut Microbiota–Kiwifruit Interactions
Source: Microorganisms. 2020 Oct 14;8(10):1582. doi: 10.3390/microorganisms8101582 (PMC7602194; doi:10.3390/microorganisms8101582)
Supplement: Supplementary file 1 [file microorganisms-08-01582-s001.pdf]

microorganisms

Supplementary material

Bioaminergic Responses in an *In Vitro* System Studying Human Gut Microbiota-Kiwifruit Interactions

Shanthi G. Parkar[1]*, Carel M. H. Jobsis[1], Tania M. Trower[2], Janine M. Cooney[2], Duncan I. Hedderley[1] and Kerry L. Bentley-Hewitt[1]*

[1] The New Zealand Institute for Plant and Food Research Limited (Plant & Food Research), Private Bag 11600, Palmerston North 4442, New Zealand; (shanthi.parkar@plantandfood.co.nz (S.G.P.); carel.jobsis@plantandfood.co.nz (C.M.H.J.); duncan.hedderley@plantandfood.co.nz (D.I.H.); kerry.bentley-hewitt@plantandfood.co.nz (K.L.B-H.).

[2] Plant & Food Research, Private Bag 11600, Hamilton 3240, New Zealand; tania.trower@plantandfood.co.nz (T.M.T.); janine.cooney@plantandfood.co.nz (J.M.C.)

* Correspondence: shanthi.parkar@plantandfood.co.nz ; shanthi.parkar@gmail.com (S.G.P.); kerry.bentley-hewitt@plantandfood.co.nz (K.L.B-H.)

2. Materials and Methods

2.3. Biogenic amine (BA) Analysis

A wide range of biogenic amines (BAs) were analyzed (Figure S1).

Standards and internal standards used included 3,4-dihydroxyphenyl acetic acid (DOPAC), 3,4 dihydroxyphenylalanine (L-DOPA), homovanillic acid (HVA), dopamine hydrochloride (DA), DL-normetanephrine HCL (NM), norepinephrine bitartrate (NE), epinephrine bitartrate (E), DL-5-hydroxytryptophan (5HTP), 5-hydroxyindole-3-acetic acid (5HIAA), DL-tyrosine (TYR), DL-metanephrine HCL (MN), DL-4-hydroxy-3-methoxymandelic acid (VMA), and gamma-aminobutyric acid (GABA), purchased from Sigma–Aldrich (St Louis, MO). Phenylethylamine (PEA) was purchased from Acros Organics (Thermo Fisher Scientific, New Jersey, US), and 3-methoxy-p-tyramine HCL (3MT), rac 3,4-dihydroxyphenylglycol (DHPG), 3-methoxy-4-hydroxyphenylglycol (MHPG), d3-homovanillic acid (d3-HVA), rac normetanephrine-d3-HCL (d3-NM), DL-norepinephrine-d6 HCL (d6-NE), rac epinephrine-d3 (d3-E), 5-hydroxyindole-3-acetic acid-d5 (d5-5HIAA), rac 3,4-dihydroxyphenylethylene glycol-d5 (d5-DHPG), rac metanephrine-d3 HCL salt (d3-MN), , 4-hydroxy-3-methoxymandelic acid-d3 (d3-VMA) and gamma-aminobutyric acid-d6 (d6-GABA), L-tyrosine-d4 (d4-TYR) were purchased from Toronto Research Chemicals (TRC, Toronto, Canada); d4-Dopamine hydrochloride (d4-DA) and 3,4 dihydroxyphenylalanine-d3 (L-DOPA-d3) were purchased from CDN Isotopes (Quebec, Canada). ReagentPlus grade acetic anhydride (AA) (≥99%), acetic anhydride-d6 (AA-d6), 99 atom % D and ReagentPlus grade 2,2,2-trifluoroethanol (TFE) (≥99%) were also purchased from Sigma–Aldrich. 2,2,2-trifluoroethanol-d2 (D, 98%) was purchased from Cambridge Isotope Laboratories, (Andover, USA) and sodium bicarbonate and sodium carbonate (anhydrous) Analar grade from BDH (King of Prussia, PA, USA).

This method uses 1H/2H6-acetic anhydride and 1H/2H2 -2,2,2-trifluoroethanol to quantitatively convert the BAs to their corresponding acetate or ester to increase their analysis sensitivity. Isotope label coding is enabled using d6-acetic anhydride and d2-2,2,2-trifluoroethanol to create an internal standard (IS X-DP) for each BA.

Stock solutions of the 21 BAs and 17 deuterated BAs were individually prepared in 50% aqueous acetonitrile plus 1% formic acid at a concentration of 1 mg/mL, with the exception of tyrosine, and d4-tyrosine (0.35 mg/mL).

Preparation of IS dX: A mixed standard solution comprising 100 ng/mL of each of the 17 deuterated BAs was prepared by diluting the appropriate volume of each stock solution with 50% aqueous acetonitrile plus 1% formic acid.

Preparation of STD-P: A mixed standard solution comprising 40 µg/mL of each of the 21 BAs and 17 deuterated BAs (dX) was made by diluting the appropriate volume of each stock solution with 50% aqueous acetonitrile plus 1% formic acid followed by derivatization with acetic anhydride and trifluoroethanol (TFE) (Figure S2a). The derivatization workflow is as described for the samples below but with the omission of the Phree plate clean-up step.

Preparation of IS X-DP: A mixed standard solution comprising 40 µg/mL of each of the 21 BAs and 17 deuterated BAs (dX) was made by diluting the appropriate volume of each stock solution with 50% aqueous acetonitrile plus 1% formic acid followed by derivatization with d6-acetic anhydride and d2-trifluoroethanol (Figure S2b). The derivatization workflow is as described for the samples below but with the omission of the Phree plate clean-up step.

Preparation of calibration standards: Calibration standards were prepared from STD-P to give concentrations of 0.02 to 1000 ng/mL. A fixed volume of IS X-DP (100 µL) was added to each calibration standard.

The samples i.e. kiwifruit puree, digesta and fermenta (100 µL) were each added to an individual well of a Phree™ phospholipid removal 96-Well Plate (Phenomenex, Torrance, CA, USA) followed by the addition of labelled internal standard (IS dX) (20 µL) and 25% acetic anhydride in acetonitrile (400 µL). As depicted in Figure S3, the mixture was vortexed (1000 rpm) for 5 min and the Phree 96-Well Plate was placed on top of a 96 Multi-Tier Micro Plate System (TOPAS) containing 2 mL conical bottom glass vials (J.G Finneran Associates, Inc., Vineland, NJ 08360 USA) for collection of the filtrate. A positive pressure Manifold (Waters, Milfor, MS, USA) set at 2 to 5 psi was applied to the plate to elute the supernatant. An additional 100 µL water in 400 µL 25% acetic anhydride in acetonitrile (v/v) was added to the Phree plate, followed by positive pressure elution. Bicarbonate-carbonate buffer (200 µL; 0.7644 g sodium bicarbonate and 0.0954 g of sodium carbonate (anhydrous) in 100 mL water) was added to the combined collected filtrate and the mixture was evaporated to dryness using a CentraVap® Refrigerated Centrifugal Concentrator, (10°C), (Labconco, Kansas City, USA). Acetic anhydride (25 µL) and TFE (75 µL) were then added to each sample well and the TOPAS system was sealed with a Molded PTFE/Silicone Mat, 96 Plugs (JG Finneran), and heated at 80°C with agitation using a ThermoMixer® C (Eppendorf, Hamburg, Germany) at 300 rpm for 6 h, then 50°C for a further 18 h before removing the seal and evaporating to dryness using the CentraVap® Refrigerated Centrifugal Concentrator, (10°C). Samples were re-derivatized with acetic anhydride (50 µL) and heated at 100°C with agitation using a ThermoMixer® C at 500 rpm for 2.5 h. Then to each sample was added IS X-DP (100 µL), acetonitrile (100 µL) and aqueous ammonium formate (250 µL; 0.06% ammonium formate in water pH adjusted to 4.8 with formic acid). Samples were vortex mixed prior to filtration through a 0.7-µm GF 96 well plate filter, and the filtrate collected in a 2-mL 96-deepwell plate (Phenomenex, Torrance, CA, USA). An aliquot (5 µL) was injected for LC-MS.

LC-MS experiments were carried out on a 5500 QTrap triple quadrupole/linear ion trap (QqLIT) mass spectrometer equipped with a Turbo V™ ion source and electrospray source (ESI) probe (AB Sciex, Concord, ON, Canada) coupled to an Ultimate 3000 UHPLC (Dionex, Sunnyvale, CA, USA). Chromatographic separation was performed on an Acquity UPLC® CSH™ C18 (2.1 x 150 mm, 1.7 µm) column (Waters, Dublin, Ireland), using 5% acetonitrile 95% (0.06% ammonium formate, pH adjusted to 4.8 with formic acid) (solvent A) and acetonitrile (solvent B) as the mobile phase for

gradient elution. The column flow rate was 0.4 mL min-1; the column temperature was 65°C, and the autosampler was kept at 5°C. The initial mobile phase, 0% B, was held for 1 min, then ramped linearly to 10% B at 6 min, held for 1 min, then 20% B at 13 min, held for 2 min, then 25% B at 20 min, 45% B at 20.5 min, 50% B at 21 min, 57% B at 23 min, 90% B between 23.5 to 30 min before resetting to the original conditions.

MS data were acquired in the positive mode using a multiple reaction monitoring method using Analyst 1.6 software and was processed using MultiQuant 3.0.2 software (AB Sciex, Concord, ON, Canada). Quantitation used the internal standard ratio method (Figure S4).

The transitions were monitored (Q1 and Q3) are listed in Table S1. Other operating parameters were as following: ion spray voltage 2500 V; temperature 700°C; curtain gas 50 psi; ion source gas 1 40 psi; ion source gas 250 psi; collision gas set to medium.

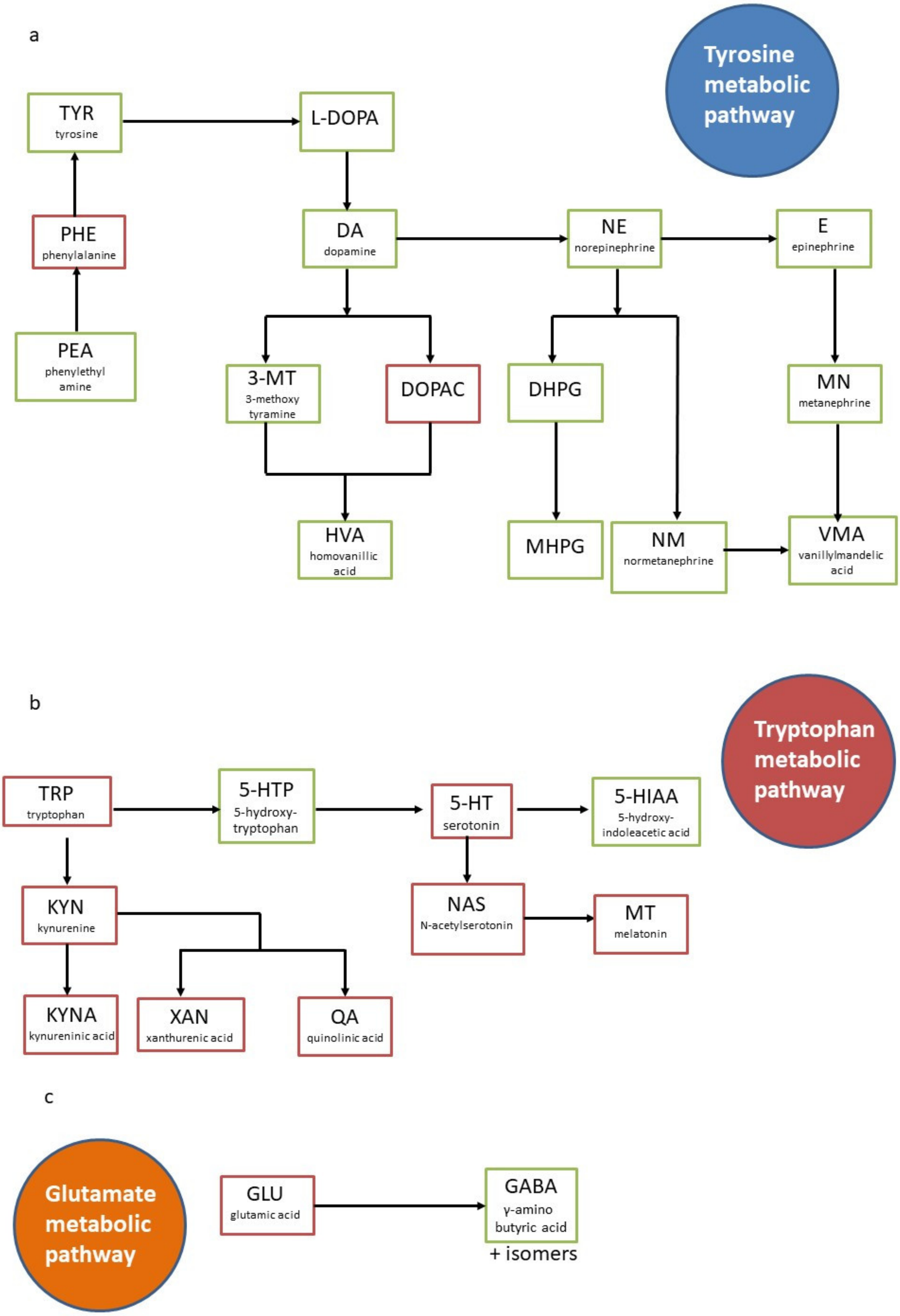

Figure S1. Schematic outlining the biogenic amines generated by the tryptophan, tyrosine and the glutamate metabolism pathways (panels a, b and c respectively). The bioamines highlighted in green

were analyzed in this study, while substrates highlighted in blue have been shown to cross the blood-brain barrier [1-4]. *Systemic GABA potentiates enteric vagal stimulation of the brain to enhance GABAergic neuronal pathways, and may potentially cross the blood-brain barrier.

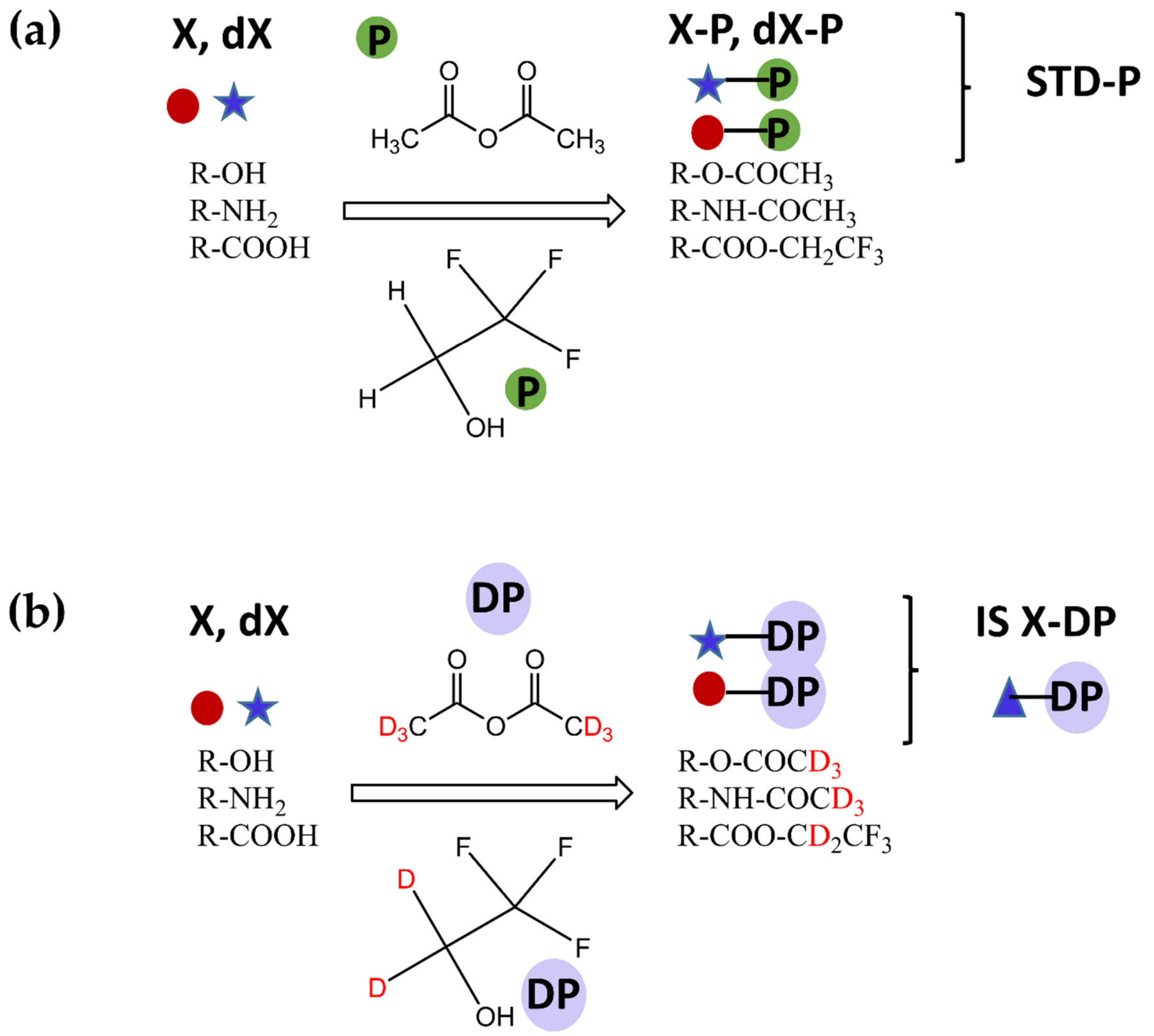

Figure S2. The derivatization schematic for the LC-MS method to analyze biogenic amines. Panel a. Derivatization of analytes (X) and labelled internal standard (dX) with probes (P). Panel b. Preparation of isotope coding labelled probe internal standard (dX) with deuterated probes (DP).

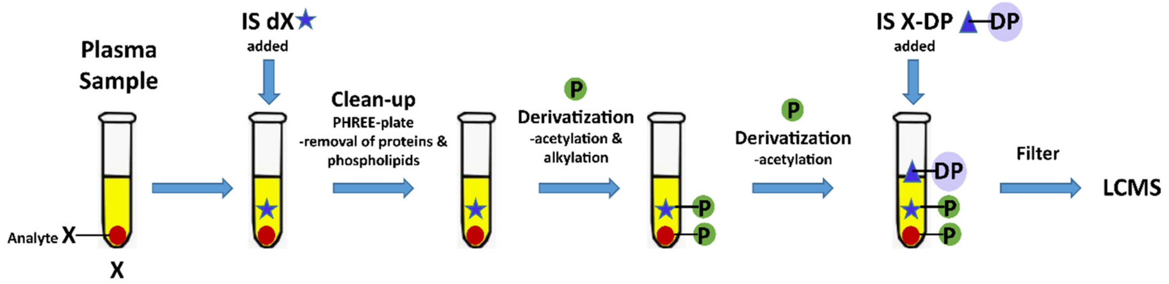

Figure S3. Workflow prior to LC-MS analysis of biogenic amines.

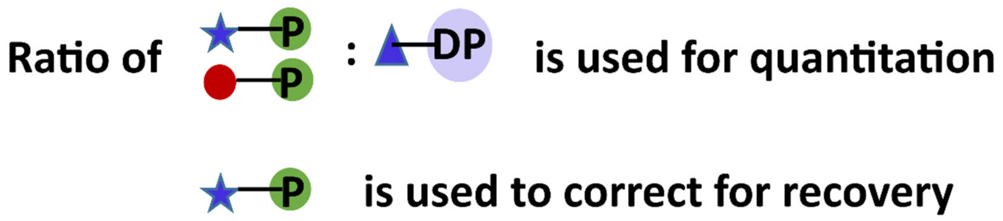

Figure S4. Transitions monitored during LC-MS quantitation of biogenic amines

Table S1. Multiple Reaction Monitoring (MRM) transitions used for biogenic amine analysis.

Q1	Q3	RT	Compound	DP	EP	CE	CXP
228	85.9		P-GABA	43.6	8	17.6	11.3
228	127		P-GABA	47	5.8	37.9	10.7
234	91.9	8.45	P-d_6-GABA	45	6	20	11
234	131	8.45	P-d_6- GABA	50	6.8	41	11.2
233	86.9	8.46	DP-GABA	53	14	20.9	10.6
239	93	8.36	DP-d_6- GABA	45	6	20	11
300[#]	181	6.50	P-VMA	55	10.9	18.5	17
300[#]	223	6.50	P-VMA	49	10.9	11.3	17
303.1[#]	149	6.46	P-d_3-VMA	20	11.3	43	13.3
303.1[#]	184	6.46	P-d_3-VMA	33	8.4	19	16.2
306[#]	182	6.41	DP-VMA	55	10.9	18.5	17
309[#]	185	6.41	DP-d_3-VMA	33	8.4	19	16.2
164	105	9.34	P-PEA	30	10	25	10
164.001	105	9.34	P-PEA - low CE	30	10	12	10

Q1	Q3	RT	Compound	DP	EP	CE	CXP
167	105	9.3	DP-PEA	30	10	25	10
280	137	10.72	P-DA	70	6.14	35	15
284.01	141.01	10.65	P-d_4-DA	30	6.9	36.2	14
301#	141	10.65	P-d_4-DA	30	4.8	47	14
289	139	10.61	DP-DA	70	6.14	35	15
293	143	10.55	DP-d_4-DA	70	10	37	15
252	91	10.81	P-3MT	27	5.1	56	11
252	210.2	10.81	P-3MT	54	7.2	16	16
258	91	10.73	DP-3MT	27	5.1	56	11
258	214	10.73	DP-3MT	54	7.2	16	16
355#	194	11.95	P-NE	10	10	30	1
361#	200	11.88	P-d_6-NE	50	10	37	17.4
361#	284	11.88	P-d_6-NE	48	5.6	15.2	8
367#	199	11.81	DP-NE	10	10	30	1
373#	293	11.74	DP-d_6-NE	48	5.6	15.2	8
373.01#	205.2	11.74	DP-d_6-NE	50	10	37	17.4
250@	166	11.97	P-NM	50	9	25	15
327#	166	11.97	P-NM	38	5.4	39.2	14.5
327#	250.1	11.97	P-NM	38	7.7	14.3	22.8
330#	169.1	11.92	P-d_3-NM	64	13.6	36.2	19.3
330#	253	11.92	P-d_3-NM	34	9.9	13.1	7.2
256@	168	11.85	DP-NM	50	9	25	15

Q1	Q3	RT	Compound	DP	EP	CE	CXP
339[#]	171.3	11.81	DP-d_3- NM	64	13.6	36.2	19.3
339[#]	259	11.81	DP-d_3-NM	34	9.9	13.1	7.2
292[@]	250	14.45	P-E	170	10	20	1
355	211	14.38	P-d_3-E	58	12.1	34	17.9
355	295	14.38	P-d_3-E	56	11.7	14.8	8.7
301[@]	257	14.31	DP-E	170	10	20	1
367	304	14.24	DP-d_3-E	56	11.7	14.8	8.7
324	180	14.57	P-MN	24	10	35	17.9
324	264	14.57	P-MN	40	11.8	13.2	22
327	183.1	14.52	P-d_3-MN	49	12.6	34.4	13.8
327	267	14.52	P-d_3-MN	50	12.3	15.4	8
333	182	14.45	DP-MN	24	10	35	17.9
333	270	14.45	DP-MN	40	11.8	13.2	22
336	229	14.39	DP-d_3-MN	50	13	26	16.7
336	273	14.39	DP-d_3-MN	50	12.3	15.4	8
356[#]	195	19.18	P-DHPG	34	6.4	30	5.98
356[#]	237	19.18	P-DHPG	34	6.3	20.7	6.68
356[#]	279	19.18	P-DHPG	34	6.4	15.4	8.2
361[#]	242	19.07	P-d_5-DHPG	27	11.1	21	6.5
361.01[#]	200.01	19.07	P-d_5-DHPG	16.4	10.8	31	19
368[#]	244	18.95	DP-DHPG	90	13	20	20
373[#]	205.2	18.83	DP-d_5- DHPG	16.4	10.8	31	19

Q1	Q3	RT	Compound	DP	EP	CE	CXP
373#	249.1	18.83	DP-d_5- DHPG	90	13	20	20
328.2#	209	19.56	P-MHPG	0	6.9	20	6.1
328.2#	250.9	19.56	P-MHPG	0	12.7	12.71	7
337#	213	19.35	DP-MHPG	0	6.9	20	6.1
337#	257	19.35	DP-MHPG	0	12.7	12.71	7
387.1	217	19.44	P-5HTP	94	11.9	31	6.1
387.1	345.1	19.44	P-5HTP	94	6.5	18	9.6
348.1	136	19.63	P-TYR	95	6.5	45	12.9
348.1	178	19.63	P-TYR	95	5.4	32	16.7
348.101	136	19.63	P-TYR - low CE	95	6.5	10	12.9
348.101	178	19.63	P-TYR - low CE	95	5.4	8	16.7
352.1	94	19.54	P-d_4-TYR	67	7.6	71.9	9.1
352.1	182.1	19.54	P-d_4-TYR	62	6.7	32.3	5.3
356	138	19.47	DP-TYR	95	6.5	45	12.9
360	95	19.38	DP-d_4-TYR	67	7.6	71.9	9.1
360	142	19.38	DP-d_4-TYR	95	6.5	45	12.9
360	186.3	19.38	DP-d_4-TYR	62	6.7	32.3	5.3
406	152	19.99	P-L-DOPA	151	6.9	51.3	13.1
423#	194	19.99	P-L-DOPA	47.8	9.2	45.7	13.6
409	197	19.93	P-d_3-L-DOPA	130	12.2	40.7	17.2
426#	197	19.93	P-d_3-L-DOPA	53	12	43.9	14.7
417	155	19.79	DP-L-DOPA	151	6.9	51.3	13.1

Q1	Q3	RT	Compound	DP	EP	CE	CXP
434#	199	19.79	DP-L-DOPA	47.8	9.2	45.7	13.6
437#	201	19.73	DP-d_3-L-DOPA	53	12	43.9	14.7
333.1#	146.1	22.19	P-5HIAA	59	6.3	41	12.7
333.1#	274.1	22.19	P-5HIAA	69	5.38	22	7.7
338.1#	279	22.17	P-d_5-5HIAA	36	7.1	22.4	8.6
338.1#	321	22.17	P-d_5-5HIAA	52	6.2	13.3	8.9
338#	146.9	22.17	DP-5HIAA	41.5	5.5	41.4	14.16
338#	277	22.17	DP-5HIAA	40	7.5	21.4	4
343#	282	22.16	DP-d_5-5HIAA	36	7.1	22.4	8.6
343#	326	22.16	DP-d_5-5HIAA	52	6.2	13.3	8.9
324.2#	206.9	22.52	P-HVA	31	5.5	14.8	19.7
324.2#	307.1	22.52	P-HVA	31	7.6	12.7	8.5
327#	210.1	22.51	P-d_3-HVA	58	4.8	16.1	2.8
327#	310.1	22.51	P-d_3-HVA	43	6.8	12	25.3
329.01#	210	22.5	DP-HVA	46	7	16.27	6.13
332.01#	213	22.49	DP-d_3-HVA	62	7	18.55	6.16
332.01#	315	22.49	DP-d_3-HVA	62	6.46	13.4	8.98

Q1 and Q3 transitions, retention time (RT), declustering potential (DP), entrance potential (EP), collision energy (CE) and collision cell exit potential (CXP) for each of the derivatized bioamines analyzed. Low CE denotes the transition has been detuned from optimum to reduce sensitivity. # denotes an ammonium adduct. @ denotes a daughter ion.

2.4. Organic acid (OA) Analysis

Standards of 14 straight-chain and branched-chain organic and short chain fatty acids (C1 through to C7), including formic acid (FA), lactic acid (LA), acetic acid (AA), propionic acid (PA), isobutyric acid (IBA), butyric acid (ButA), succinic acid (SucA), 2-methyl butyric acid (2MBA), isovaleric acid (IVA), valeric acid (VA), 3-methyl valeric acid (3MVA), isocaproic acid (ICA), caproic acid (CA) and

heptanoic acid (HA) were purchased from Sigma–Aldrich (St Louis, MO). Analytical reagent-grade 3-nitrophenylhydrazine (3NPH). HCl (97%) was also purchased from Sigma–Aldrich. N-(3-dimethylaminopropyl)-NO-ethylcarbodiimide (EDC). HCl was purchased from MP Biochemicals Inc. (Thermo Fisher Scientific, Waltham, MA, USA) and 13C6-3NPH. HCl from IsoSciences Inc. (King of Prussia, PA, USA).

This method uses 12C/13C6-3-nitrophenylhydrazine (3NPH) to quantitatively convert OAs to their 3-nitrophenylhydrazones to increase their analysis sensitivity. Isotope label coding is enabled using 13C6-3NPH to create an internal standard (IS) for each OA.

Stock solutions of the 14 OAs were individually prepared in 50% aqueous acetonitrile at a concentration of 10 mg/mL. A mixed standard solution containing 1 mM of each of the 14 OAs was made by diluting the appropriate volume of each stock solution with 50% aqueous acetonitrile. This solution was further diluted to prepare standards of concentrations of 250 μM to 0.05 μM. To cover the linear dynamic range of the more abundant OAs (FA, AA, PA, ButA, LA, SucA), additional working standards were prepared from the stock solutions at the following concentrations: 40 mM (AA, PA, ButA), 20 mM, 10mM, 5mM and 2mM (FA, AA, PA, ButA, LA, SucA).

Fifty microliters of a mixed standard solution containing 4 mM of FA, AA and HA, 2 mM of PA and LA, and 1 mM of each of the other nine OAs were added to a 2-mL safelock Eppendorf tube that contained 1 mg of 13C6-3NPH.HCl. To this was added EDC-6% pyridine (25 μL; 120 mM in methanol) and 75:25 methanol/water v/v (25 μL). The mixture was reacted at 4°C overnight. The reaction was quenched by the addition of quinic acid (25 μL; 200 mM in 75:25 methanol/water v/v), and the solution transferred to a volumetric flask with 10% aqueous methanol and diluted with the same solvent to 100 mL. This solution was used as the IS mix. This solution was stable when stored at -20°C.

LC-MS experiments were carried out on a 5500 QTrap triple quadrupole/linear ion trap (QqLIT) mass spectrometer equipped with a Turbo V™ ion source and atmospheric pressure chemical ionisation (APCI) probe (AB Sciex, Concord, ON, Canada) coupled to an Ultimate 3000 UHPLC (Dionex, Sunnyvale, CA, USA). Chromatographic separation was performed on an Acquity UPLC® CSH™ C18 (2.1 x 150 mm, 1.7 μm) column (Waters, Dublin, Ireland), using water (solvent A) and acetonitrile (solvent B) as the mobile phase for gradient elution. The column flow rate was 0.4 mL min-1; the column temperature was 65°C, and the autosampler was kept at 5°C. The initial mobile phase, 0.5% B, was ramped linearly to 2.5% B at 3 min, then 17% B at 6 min, 45% B at 10 min, 55% B at 13 min, 100% B between 14 and 18 min before resetting to the original conditions.

MS data were acquired in the negative mode using a multiple reaction monitoring method using Analyst 1.6 software and was processed using MultiQuant 3.0.2 software (AB Sciex, Concord, ON, Canada).

The transitions monitored (Q1 and Q3) are listed in Table S2. Extra transitions for the more abundant OAs were detuned from optimum to reduce their sensitivity to fit within the dynamic linear range of the instrument (denoted in Table S2 as low CE). Other operating parameters were as following: ion spray voltage -4500 V; temperature 420°C; curtain gas 50 psi; ion source gas 1 50 psi; ion source gas 2 70 psi; collision gas set to medium.

Table S2. Multiple Reaction Monitoring transitions used for organic acid analysis.

Q1	Q3	RT	Compound	DP	EP	CE	CXP
326.1	137	6.62	QA-3NPH low CE	-250	-10	-20	-15

Q1	Q3	RT	Compound	DP	EP	CE	CXP
180.2	106.8	6.78	FA-3NPH	-43.5	-7.1	-27.26	-10.3
180.2	136.8	6.78	FA-3NPH	-59.7	-6.56	-18.69	-11.59
180.201	106.8	6.78	FA-3NPH low CE	-43.5	-7.1	-11	-10.3
186.2	142.8	6.78	FA-3NPH$^{13}C_6$	-59.7	-6.56	-18.69	-11.59
223.9	136.7	7.22	LA-3NPH	-70	-6.06	-40	-15
223.9	151.8	7.22	LA-3NPH	-49.6	-6.5	-19.6	-14.7
223.901	136.701	7.22	LA-3NPH low CE	-70	-6.06	-15	-15
223.902	136.702	7.22	LA-3NPH low CE	-70	-6.06	-11	-15
229.9	157.8	7.22	LA-3NPH$^{13}C_6$	-49.6	-6.5	-19.6	-14.7
194.2	136.6	7.34	AA-3NPH	-43	-6.8	-24.02	-10.9
194.2	152	7.34	AA-3NPH	-54.77	-6.5	-17.13	-12.14
194.201	152.001	7.34	AA-3NPH low CE	-54.77	-6.5	-12	-12.14
200.2	158	7.34	AA-3NPH$^{13}C_6$	-54.77	-6.5	-17.13	-12.14
208	136.8	8.55	PA-3NPH	-80	-4	-25	-11
208	165	8.55	PA-3NPH	-70	-5.1	-18.97	-13.91
208.001	165	8.55	PA-3NPH low CE	-70	-5.1	-6	-13.91
214	142.8	8.55	PA-3NPH$^{13}C_6$	-80	-4	-25	-11
222.2	136.7	9.46	IBA-3NPH	-91.55	-5.92	-25.8	-11
222.2	179	9.46	IBA-3NPH	-23.31	-2.19	-19.49	-15.01
228.2	142.7	9.46	IBA-3NPH$^{13}C_6$	-91.55	-5.92	-25.8	-11
222.201	136.7	9.58	ButA-3NPH	-17	-4.7	-25.86	-12
222.201	179	9.58	ButA-3NPH	-57.9	-5.2	-18	-14

Q1	Q3	RT	Compound	DP	EP	CE	CXP
222.202	179	9.58	ButA-3NPH low CE	-57.9	-5.2	-7	-14
228.201	142.7	9.58	ButA-3NPH$^{13}C_6$	-17	-4.7	-25.86	-12
387.1	137	9.95	SucA-3NPH	-39	-6.79	-38.8	-11.9
387.1	234	9.95	SucA-3NPH	-23	-6.28	-25	-20
387.101	137	9.95	SucA-3NPH low CE	-39	-6.79	-21	-11.9
399.1	240	9.95	SucA-3NPH$^{13}C_6$	-23	-6.28	-25	-20
236.3	107	10.32	2MBA-3NPH	-75	-6.6	-36	-9.5
236.3	136.7	10.32	2MBA-3NPH	-75	-6.6	-28	-10.5
242.3	142.7	10.32	2MBA-3NPH$^{13}C_6$	-75	-6.6	-28	-10.5
236.1	106.7	10.42	IVA-3NPH	-2.21	-6.3	-36.3	-2.8
236.1	137	10.42	IVA-3NPH	-98	-5.6	-24.8	-12.5
242.1	143	10.42	IVA-3NPH$^{13}C_6$	-98	-5.6	-24.8	-12.5
236.1	136.7	10.57	VA-3NPH	-13.58	-4.21	-26	-17.78
236.1	152	10.57	VA-3NPH	-13.46	-5.3	-23.12	-13
242.1	142.7	10.57	VA-3NPH$^{13}C_6$	-13.58	-4.21	-26	-17.78
250.201	137	11.23	3MVA-3NPH	-80	-4.5	-28.5	-12
250.201	152	11.23	3MVA-3NPH	-57	-5	-23.5	-11.1
256.201	143	11.23	3MVA-3NPH$^{13}C_6$	-80	-4.5	-28.5	-12
250.2	136.8	11.32	4MVA-3NPH	-95	-4.2	-28	-11.2
250.2	151.9	11.32	4MVA-3NPH	-76	-3.4	-23.4	-11.6
256.2	142.8	11.32	4MVA-3NPH$^{13}C_6$	-95	-4.2	-28	-11.2
250.2	136.9	11.44	CA-3NPH	-75	-5.1	-30	-12.4

Q1	Q3	RT	Compound	DP	EP	CE	CXP
250.2	152	11.44	CA-3NPH	-77	-5.2	-24.3	-12.86
256.2	142.9	11.44	CA-3NPH$^{13}C_6$	-75	-5.1	-30	-12.4
264.1	107	12.35	HA-3NPH	-79	-12	-39.8	-10
264.1	137	12.35	HA-3NPH	-83	-10.5	-31.5	-12.1
270.1	143	12.35	HA-3NPH$^{13}C_6$	-83	-10.5	-31.5	-12.1

Q1 and Q3 transitions, retention time (RT), declustering potential (DP), entrance potential (EP), collision energy (CE) and collision cell exit potential (CXP) for each of the derivatized organic acids analyzed. Low CE denotes the transition has been detuned from optimum to reduce sensitivity.

2.5. *DNA Extraction, Microbiome Characterization and Bioinformatics*

The inoculum i.e. the pooled fecal slurry and the endpoint of the fermentation, i.e. 18-h fermenta, were used for the extraction of the DNA. Three 1-mL aliquots of the pooled fecal slurry and duplicate 1-mL aliquots of each 18-h fermenta were processed separately. Samples were centrifuged at 17,000 x g for 5 min at 4°C. DNA was extracted from pellets using the DNeasy PowerLyzer PowerSoil Kit (QIAGEN Pty Ltd, Victoria, Australia) as per the manufacturer's instructions, with homogenization performed in a Fastprep-24 5G (MP Biomedicals, Irvine, CA) at 4.0 m/s for 45 s. The duplicate fermenta DNAs were pooled then processed with Genomic DNA Clean & Concentrator-10 kit (Zymo Research, Orange, CA, USA) as per the manufacturer's instructions, and eluted in 40 μL of PowerSoil C6 solution. DNA quantity and purity were measured on a QIAxpert (QIAGEN Pty Ltd, Victoria, Australia).

DNA was submitted to the Massey Genome Service (Palmerston North, New Zealand) for dual-indexing sequencing on the Illumina® MiSeq Sequencing platform. First, a PCR was run using Invitrogen AccuPrime™ Pfx SuperMix (Cat—12344-040) (17 μL), 10 μM 16SR_V4 Primer (1 μL), 10 μM 16SF_V3 Primer (1 μL) and 1 μL normalized DNA sample (5 ng/μL), to amplify variable regions V3-V4 of the 16S rRNA gene of the bacterial DNA. The primers used were 16SF_V3 (5′-AATGATACGGCGACCACCGAGATCTACAC-barcode-TATGGTAATTGGCCTACGGGAGGCAGCAG-3′) and 16SR_V4 (5′-CAAGCAGAAGACGGCATACGAGAT-barcode-AGTCAGTCAGCCGGACTACHVGGGTWTCTAAT-3′) [5], which also contained adaptors for the sequencing. The PCR started with a denaturation step at 95°C for 2 min followed by 30 cycles of 95°C for 20 s, 55°C for 15 s, and 72°C for 5 min, and then a final extension step at 72°C for 10 min, and then cooling at 4°C. The PCR product was then cleaned up, normalized and pooled using the Invitrogen SequalPrep Normalization Plate Kit (ThermoFisher Scientific, Waltham, MA, USA). A Qubit DNA High Sensitivity assay was used to measure the library concentration and a Bioanalyzer DNA High Sensitivity assay (Agilent Technologies, Santa Clara, CA, USA) was used for library sizing. The amplicons were pooled in equal molarity and 16S rRNA gene sequencing performed on 2 x 250 base paired-end run using the MiSeq Sequencer (Illumina, San Diego, CA, USA).

The Illumina amplicon sequences were analyzed using Quantitative Insights Into Microbial Ecology 2 (QIIME 2, v2019.7) [6]. The sequences were first quality checked, then de-multiplexed, following which sequencing errors and chimeras were filtered by trimming using the DADA2

package [7], after which phylogenetic placement [8], taxonomic assignment using Greengenes database (v13.8, with 99% sequence similarity) [9] were performed. The microbiome α-diversity metrics – observed orthologous taxonomic units or OTUS, Chao1, Shannon index, Simpson index were calculated using the QIIME2 workflow [6]. The between-sample β diversity was tested using Bray Curtis distance matrix and the principal co-ordinate analysis (PCoA) outputs were visualized using Emperor plots. The β-diversity biplots were generated using the relative frequency data and taxonomy data. The frequencies from the samples for the 18-h fermenta were separately analyzed in similar workflows for α- and β-diversity.

2.6. Effect of Kiwifruit on Caco-2 Cells

2.6.1. Cell culture

Caco-2 cells (human colonic adenocarcinoma cells, ATCC® HTB-37™) were used as an intestinal epithelial cell model. The cells were grown in Eagle's minimum essential medium (MEM) containing GlutaMAX, nucleosides and supplemented with additives to contain 1.5 g/L sodium bicarbonate, 0.1 mM non-essential amino acids, 1.0 mM sodium pyruvate and 10% fetal bovine serum. Antibiotics and antimycotic were also added to the medium at final concentrations of 100 units penicillin, 100 µg/mL streptomycin and 0.25 µg/mL neomycin (PSN). All cell incubations were carried out 37°C under an atmosphere of 5% CO_2.

The cell lines used were between passages 20 and 24. Fetal bovine serum was obtained from Sigma-Aldrich, Auckland, New Zealand. All other tissue culture reagents were obtained from Life Technologies, Auckland, New Zealand.

2.6.2. Cell viability assay

Caco-2 cells were seeded with 200 µL of cells at 5 x 10^4 per mL in 96-well plates. The medium was changed every 48 h and were used when the cells reached confluence. Cells were treated with the baseline, 5- and 18-h fermenta (from all the four treatments) diluted in PSN-free cell culture media at 50 and 20% dilutions and incubated for 12 h. Other treatments were 0.5 and 0.1% fecal slurry (inoculum) in bacterial culture media, with each tested at two different concentrations, i.e. 50 and 20%, diluted with cell culture media and untreated controls. All fermenta and fecal slurries were first centrifuged at 17,000 x g for 5 min and sterile-filtered through a 0.2-µm filter prior to treating the cells. Cells were then washed 3 x in Dulbecco's phosphate buffered saline (DPBS) with calcium and magnesium, followed by addition of WST-1 Reagent (Roche, Auckland, New Zealand), diluted 1:10 in cell culture media with no PSN. Cells were incubated for 30 min and absorbance measurements were read at 450 nm with FLUOStar Optima® (BMG Labtech, Victoria, Australia). Some fermenta treatments treated at 50% showed signs of cell death, whilst cells treated at 20% were similar to untreated controls and 20 % dilutions were therefore used for the gene expression assay.

2.6.2. Intestinal gene expression studies

One mL of Caco-2 cells were seeded at 5 x 10^4 per mL in 24-well plates. The medium was changed every 48 h and were used when the cells reached confluence. For 24 h before the assay, the cells were washed and grown in MEM without PSN. Cells were pre-incubated with 50% fetal bovine serum in cell culture media for 2 h, to prime all the cells to the same circadian phase [10], and then washed twice in pre-warmed Dulbecco's phosphate buffered saline with calcium and magnesium. Cells were then treated with the 0-, 5- and 18-h fermenta (20% dilutions in PSN-free cell culture media). The 0-h fermenta were tested before the addition of the fecal inoculum, to examine the effect of the digested substrates. The pooled fecal inoculum used for the fermentation was included as a separate control to test the effect of the fecal bacteria, and it was diluted in the fermentation medium before the 3-h incubation. Caco-2 cells incubated only with background cell culture medium were also included as

controls. All fermenta and fecal slurries in bacteria culture media were first centrifuged at 17,000 x g for 5 min and sterile-filtered through a 0.2-μm filter prior to treating the cells. Cells were gently washed with pre-warmed DPBS with calcium and magnesium twice. Next, 350 μL of RLT buffer (Qiagen, Germany) was added to cells and left for 5 min at room temperature. Cell lysates were then collected into DNAse/RNase-free 1.5-mL tubes and an equal volume of 70% molecular grade ethanol was added. Cell lysates were stored at -80°C prior to RNA extractions. Cell lysates were thawed and transferred to an RNeasy MINI column (Qiagen, Germany). RNA was extracted as per the manufacturer's protocol (RNeasy mini handbook, Fourth edition, June 2012) into 60 μL of water, and stored at -80°C. RNA quantity were assessed by QIAxpert (Qiagen, Germany).The RNA was used for gene expression of 24 genes by the Counter Analysis System (NanoString Technologies, USA) (Table S3).

microorganisms

MDPI

Table S3. Intestinal gene targets analyzed.

Gene Name	GenBank accession number	Target region	Target Sequence
**Glucose-6-phosphate isomerase (*GPI*)**	NM_000175.2	1696-1795	CAGTGCTCAAGTGACCTCTCACGACGCTTCTACCAATGGGCTCATCAACTTCATCAAGCAGCAGCGCGAGGCCAGAGTCCAATAAACTCGTGCTCATCTG
**Charged multivesicular body protein 2A (*CHMP2A*)**	NM_014453.3	242-341	GGAGCTGGACCGCGAGCGACAGAAACTAGAGACCCAGGAGAAGAAAATCATTGCAGACATTAAGAAGATGGCCAAGCAAGGCCAGATGGATGCTGTTCGC
**Hypoxanthine Phosphoribosyltransferase 1 (*HPRT1*)**	NM_000194.1	241-340	TGTGATGAAGGAGATGGGAGGCCATCACATTGTAGCCCTCTGTGTGCTCAAGGGGGGCTATAAATTCTTTGCTGACCTGCTGGATTACATCAAAGCACTG
**Peptidylprolyl isomerase A (*PPIA*)**	NM_021130.3	316-415	TCTATGGGGAGAAATTTGAAGATGAGAACTTCATCCTAAAGCATACGGGTCCTGGCATCTTGTCCATGGCAAATGCTGGACCCAACACAAATGGTTCCCA
**Succinate Dehydrogenase Complex Flavoprotein Subunit A (*SDHA*)**	NM_004168.1	231-330	TGGAGGGGCAGGCTTGCGAGCTGCATTTGGCCTTTCTGAGGCAGGGTTTAATACAGCATGTGTTACCAAGCTGTTTCCTACCAGGTCACACACTGTTGCA
Period 1 (*PER1*)	NM_002616.2	1919-2018	CAGCAGCTTTTTATTGAGTCTCGGGCCCGGCCTCAGTCCCGGCCCCGCCTCCCTGCTACAGGCACGTTCAAGGCCAAGGCCCTTCCCTGCCAATCCCCAG
Period 2 (*PER2*)	NM_022817.2	986-1085	TGTGGAGCATGTGCAGTGGAGCAGATTCTTTTACTCAAGAATGCATGGAGGAGAAATCTTTCTTTTGCCGTGTCAGTGTCCGGAAAAGCCACGAGAATGA
Period 3 (*PER3*)	NM_016831.1	1076-1175	GACCTGATTGGAACATCGATCCTAAGCTACCTGCACCCTGAAGATCGTTCTCTGATGGTTGCCATACACCAAAAAGTTTTGAAGTATGCAGGGCATCCTC

Circadian Locomotor Output Cycles Kaput (*CLOCK*)	NM_004898.2	2351-2450	AGCCGGAAGCATGGTCCAGATTCCATCTAGTATGCCACAAAACAGCACCCAGAGTGCTGCAGTAACTACATTCACTCAGGACAGGCAGATAAGATTTTCT
Aryl hydrocarbon receptor nuclear translocator-like protein 1 (*ARNTL*)	NM_001030272.1	841-940	GATGTGACCGAGGGAAGATACTCTTTGTCTCAGAGTCTGTCTTCAAGATCCTCAACTACAGCCAGAATGATCTGATTGGTCAGAGTTTGTTTGACTACCT
Cryptochrome Circadian Regulator 1 (*CRY1*)	NM_004075.3	1376-1475	GAAGTGATAGAAAAGTGCACAACTCCTCTGTCTGATGACCATGATGAGAAATATGGAGTCCCTTCACTGGAAGAGCTAGGTTTTGATACAGATGGCTTAT
Cryptochrome Circadian Regulator 2 (*CRY2*)	NM_001127457.1	3326-3425	ATCACACTGACAGGCTTCTTCCTGAGATATCCTCAGGTTTTCTCAGCCAGAGAGCTGCCTTTAGAGTCCAACTGTTGTACGTATGTCACCTTCACTAGAA
Melatonin receptor type 1A (*MTNR1A*)	NM_005958.3	626-725	TCATCTTCTGTTACCTGAGAATATGGATCCTGGTTCTCCAGGTCAGACAGAGGGTGAAACCTGACCGCAAACCCAAACTGAAACCACAGGACTTCAGGAA
Melatonin receptor type 1B (M*TNR1B*)	NM_005959.3	956-1055	ATCCCTGAGGGGCTATTTGTCACTAGCTACTTACTGGCTTATTTCAACAGCTGCCTGAATGCCATTGTCTATGGGCTCTTGAACCAAAACTTCCGCAGGG
N-Acetylserotonin O-methyltransferase (*ASMT*)	NM_001171039.1	81-180	AGGACCAGGCCTATCGCCTCCTTAATGACTACGCCAACGGCTTCATGGTGTCCCAGGTTCTCTTCGCCGCCTGCGAGCTGGGCGTGTTTGACCTTCTCGC
Tumor necrosis factor alpha (*TNF-alpha*)	NM_000594.2	1011-1110	AGCAACAAGACCACCACTTCGAAACCTGGGATTCAGGAATGTGTGGCCTGCACAGTGAAGTGCTGGCAACCACTAAGAATTCAAACTGGGGCCTCCAGAA
Interleukin 10 (*IL10*)	NM_000572.2	231-330	AAGGATCAGCTGGACAACTTGTTGTTAAAGGAGTCCTTGCTGGAGGACTTTAAGGGTTACCTGGGTTGCCAAGCCTTGTCTGAGATGATCCAGTTTTACC
Nitric Oxide Synthase 2 (*NOS2*)	NM_000625.4	606-705	TTGCCTGGGGTCCATTATGACTCCCAAAAGTTTGACCAGAGGACCCAGGGACAAGCCTACCCCTCCAGATGAGCTTCTACCTCAAGCTATCGAATTTGTC

Tryptophan hydroxylase 2 (*TPH2*)	NM_173353.3	393-492	TCAGGAAAAACGTGTCAACATGGTTCATATTGAATCCAGGAAATCTCGGCGAAGAAGTTCTGAGGTTGAAATCTTTGTGGACTGTGAGTGTGGGAAAACA
Tryptophan hydroxylase 1 (*TPH1*)	NM_004179.1	336-435	TTCTGACCTGGACCATTGTGCCAACAGAGTTCTGATGTATGGATCTGAACTAGATGCAGACCATCCTGGCTTCAAAGACAATGTCTACCGTAAACGTCGA
Claudin-1 (*CLDN1*)	NM_021101.3	411-510	GCAAAGTCTTTGACTCCTTGCTGAATCTGAGCAGCACATTGCAAGCAACCCGTGCCTTGATGGTGGTTGGCATCCTCCTGGGAGTGATAGCAATCTTTGT
Occludin (*OCLN*)	NM_002538.3	1976-2075	GTTGGAGACTATGATAGACAGAAAACATAGAAGGCTGATGCCAAGTTGTTTGAGAAATTAAGTATCTGACATCTCTGCAATCTTCTCAGAAGGCAAATGA
Claudin-4 (*CLDN4*)	NM_001305.3	1243-1342	GGGAGCTGGCTTCTGCTGGCCAGGATAGCTTAACCCTGACTTTGGGATCTGCCTGCATCGGCGTTGGCCACTGTCCCCATTTACATTTTCCCCACTCTGT
Solute Carrier Family 6 Member 4 (*SLC6A4*)	NM_001045.2	236-335	CCCAGAGATCAATTGGGATCCTTGGCAGATGGACATCAGTGTCATTTACTAACCAGCAGGATGGAGACGACGCCCTTGAATTCTCAGAAGCAGCTATCAG

2 The internal reference genes that were used for normalization of the target genes are given in **bold** font.

2.7. Statistical analysis

The microbiome α-diversity metrics for all the samples (fecal slurry and 18-h fermenta) were analyzed for significance using the Kruskal-Wallis test, with pairwise significance testing set at $p < 0.05$. β-diversity metrics were analyzed using permutational multivariate analysis of variance (PERMANOVA).

For differential abundance analysis of the microbiome data, the amplicon sequence variants (ASVs) that had a frequency of less than 0.1% of the mean sample depth were removed (to account for possible Illumina sequencing errors). The analysis was tested using DESeq2, with likelihood ratios tested followed by testing the 18-h samples to examine differences between each substrate. The *p* values adjusted for false discovery rate are quoted [11].

3. Results

The biogenic amines (BAs), MN, VMA and HVA were not detected in any of the samples. PEA, the precursor to the tyrosine metabolic pathway, was found to be increased by microbial fermentation although there was no significant discrimination by the treatment (data not shown). Other significant changes (shown in Figure S5) in terms of the BAs from the TYR pathway include: increase in DA in the intestinal digesta of both the kiwifruit (compared with inulin and water); increase in 3MT in fermenta of both the kiwifruit (> 2-fold) and inulin (< 2-fold) compared with water fermenta; increase in DHPG in gastric and intestinal digesta of 'Zesy002' (114-fold and 76-fold respectively) and 'Hayward' (57- and 16-fold respectively) compared with water control. 3MT, the DA breakdown product, was increased significantly in both the kiwifruit and inulin fermenta compared with the water fermenta. In the case of the tryptophan pathway, there were significant increases in the 5HT precursor, 5HTP, in the gastric and intestinal digesta of 'Zesy002' (7-fold and 2-fold respectively), 'Hayward' (14- and 4-fold respectively) and inulin (2- and 0.4-fold respectively) compared with the water control. There was also a change in the concentration of the 5HT breakdown product, 5HIAA, with a significant decrease in 'Zesy002' fermenta as compared with the control.

The microbiome composition was analyzed after sequencing of the 15 samples (three samples of the fecal inoculum, 12 of the 18-h fermenta samples). A total of 548,258 frequencies were obtained that were used to generate 523 amplicon sequence variants (ASVs). Figures S7, S8 and S9 depict the mean relative frequency of the various microbial taxa at the phylum, order and family levels respectively. The α-diversity analysis showed significant ($p < 0.05$) changes in diversity metrics in comparison with the 18-h fermentation samples; the microbial community of the fecal inoculum at 0 h showed increased observed_otus, Chao1 indices, Shannon and Simpson indices (figure not shown). Bray Curtis, unweighted and weighted PCoA plots showed significant clustering effect of the microbiome that differentiated the inoculum from the 18-h fermentations (figure not shown). The overall comparison between all the samples, including the 18-h fermenta and fecal inoculum (which one may consider as generated from an *in vivo* fermenter using a mixed diet substrate), showed a significance of $p < 0.05$, the diversity of the 18-h samples generated from the *in vitro* fermentations was analyzed separately. The α-diversity metrics, observed_otus and Chao1 index showed significance between all substrates, and in all pairwise comparisons. Shannon and Simpson indices showed significance ($p < 0.05$ for overall comparisons, but not in pairwise comparisons between substrates (Figures S10). Water fermenta showed the greatest diversity, 'Hayward' showed the least, while inulin and 'Zesy002' were intermediate. Significant substrate–specific clustering was observed with Bray Curtis PCoA biplot (Figure S11).

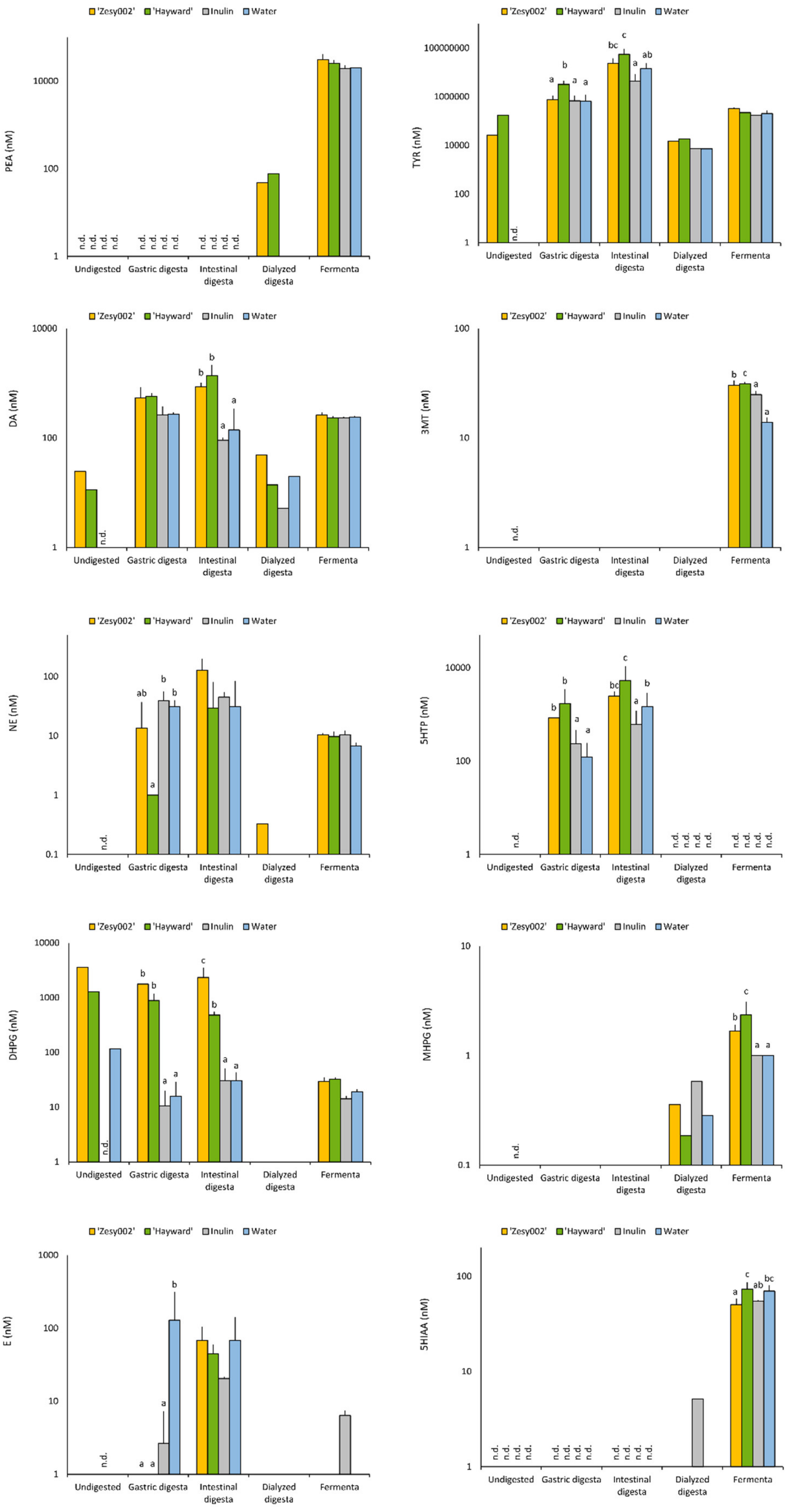

Figure S5. Concentrations of different bioamines after various stages of gastrointestinal digestion and fermentation of kiwifruit. Values are means ± standard deviation. In case of the undigested

samples, only 'Hayward', 'Zesy002' and water samples were analysed, and only singly. The dialyzed digesta were analyzed singly for all the substrates.There were three samples (n=3) for all other treatments. Within a type of digesta, means which have a letter in common are not significantly different (Tukey's LSD test (0.05)). n.d., not detectable. PEA = phenylethylamine, TYR = tyrosine, DA = dopamine, MT= 3-methoxy-p-tyramine, NE = norepinephrine, 5HTP = 5-hydroxytryptophan, DHPG = 3,4-dihydroxyphenylethylene glycol, MHPG = 3-methoxy-4-hydroxyphenylglycol, E = epinephrine, 5HIAA= 5-hydroxyindole-3-acetic acid.

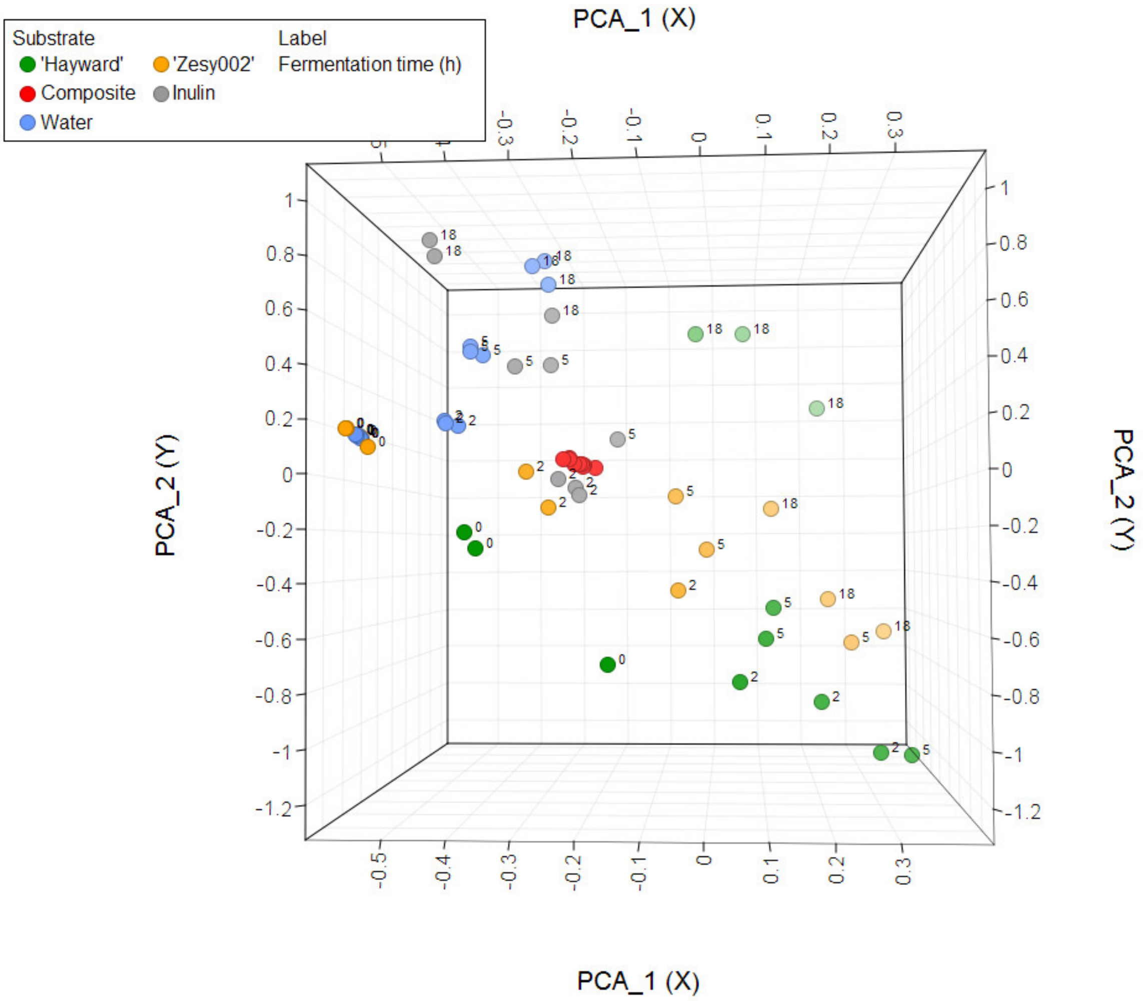

Figure S6. A principal components analysis plot showing a visualization of the influences of the substrates in generating organic acid metabolites over the duration of the fecal microbial fermentation (0, 2, 5 and 18 h). A composite sample, generated by subsampling each sample type and time, illustrates the precision of analysis.

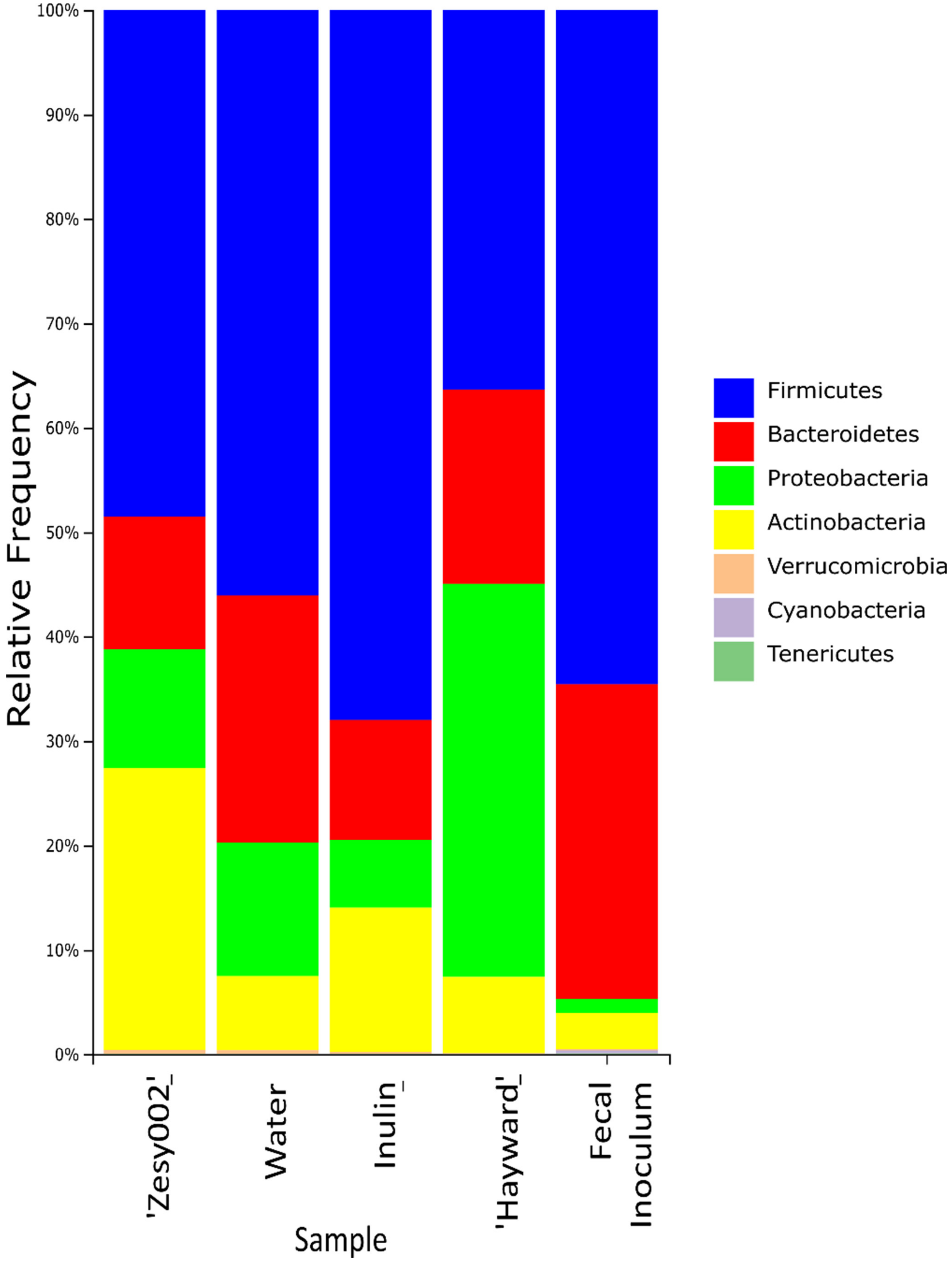

Figure S7. Mean relative frequency of microbiota of samples at phylum level.

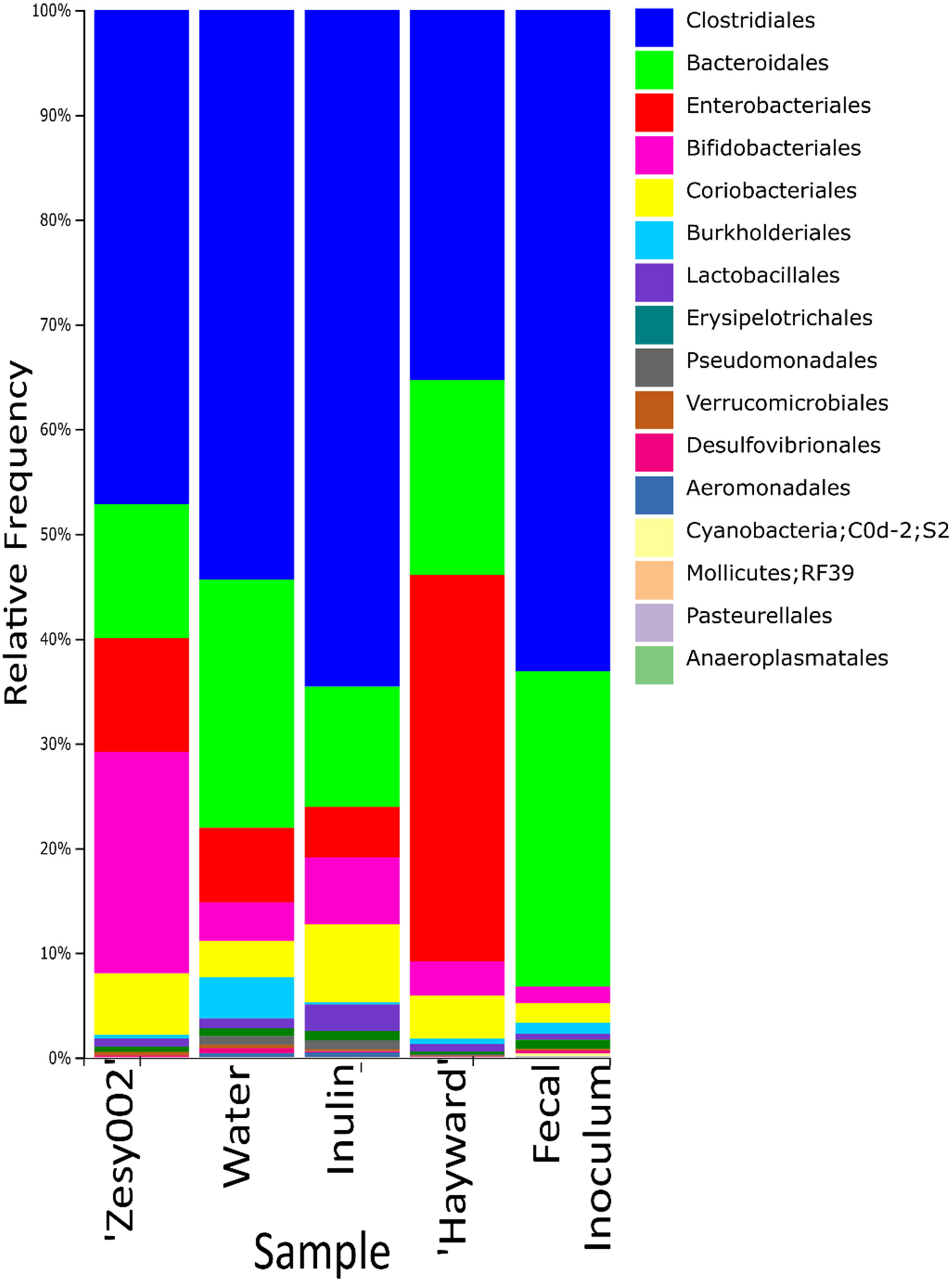

Figure S8. Mean relative frequency of microbiota of samples at order level.

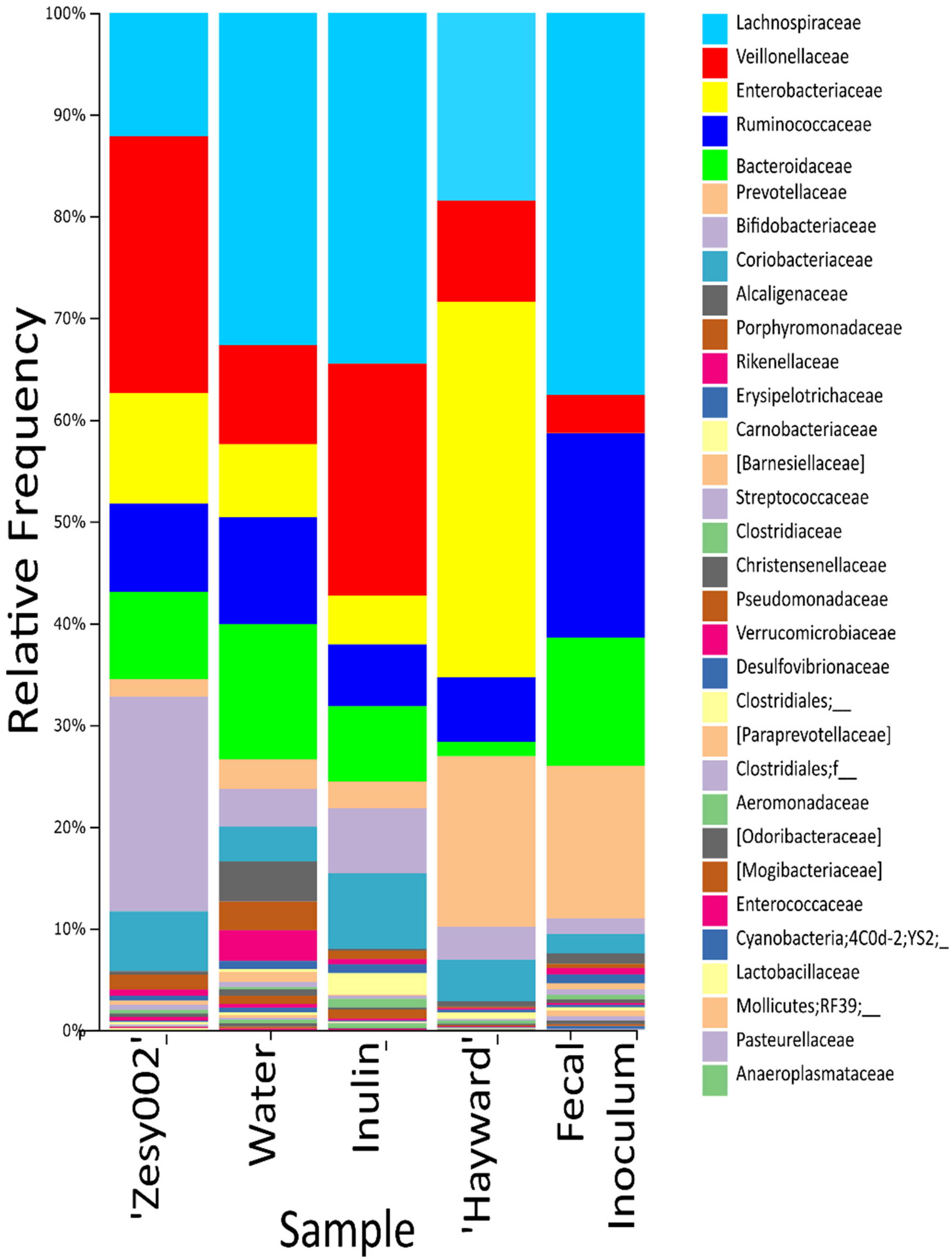

Figure S9. Mean relative frequency of microbiota of samples at family level.

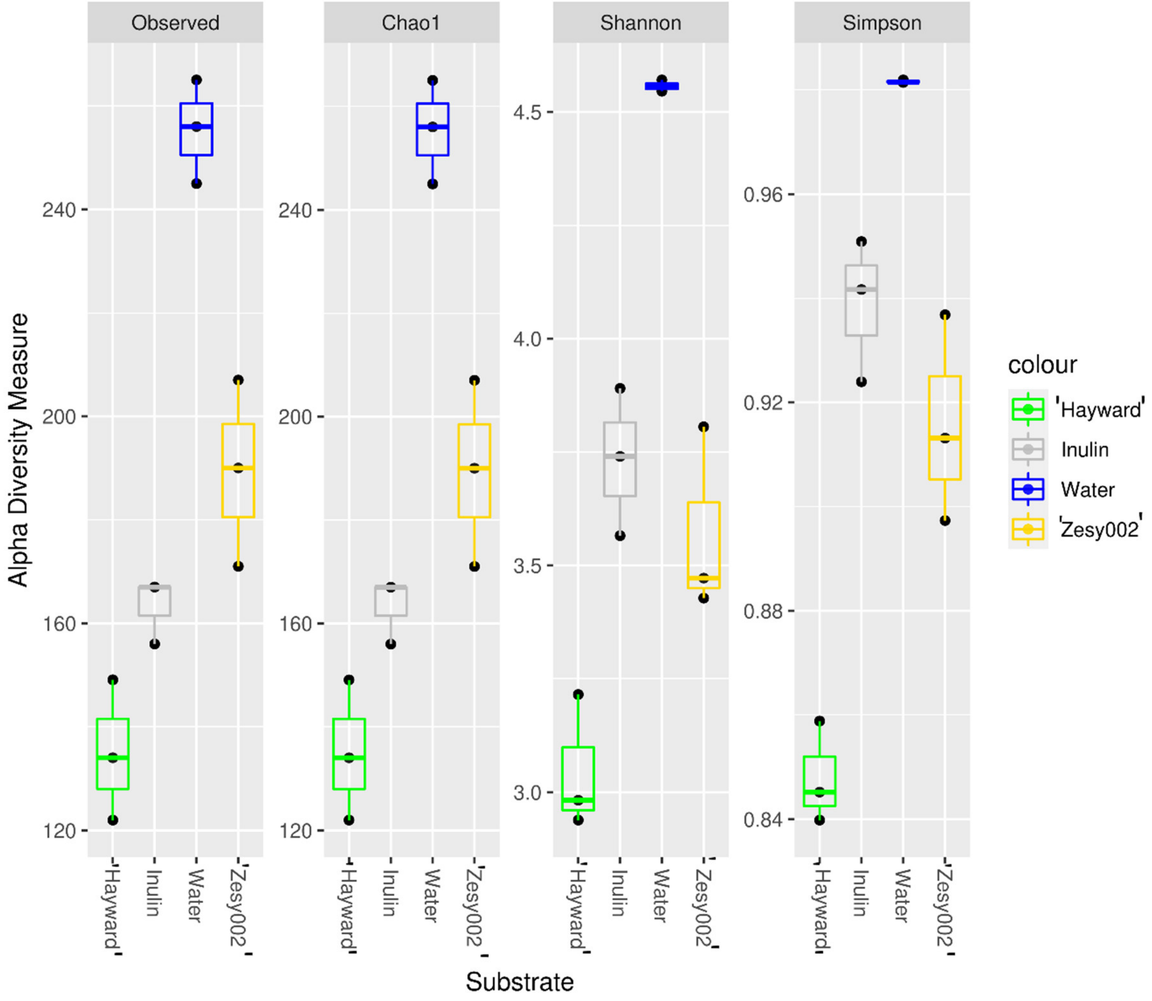

Figure S10. Diversity analysis examining changes in microbial community within samples. Significance of $p < 0.001$ for changes between all the substrates with all the metrics.

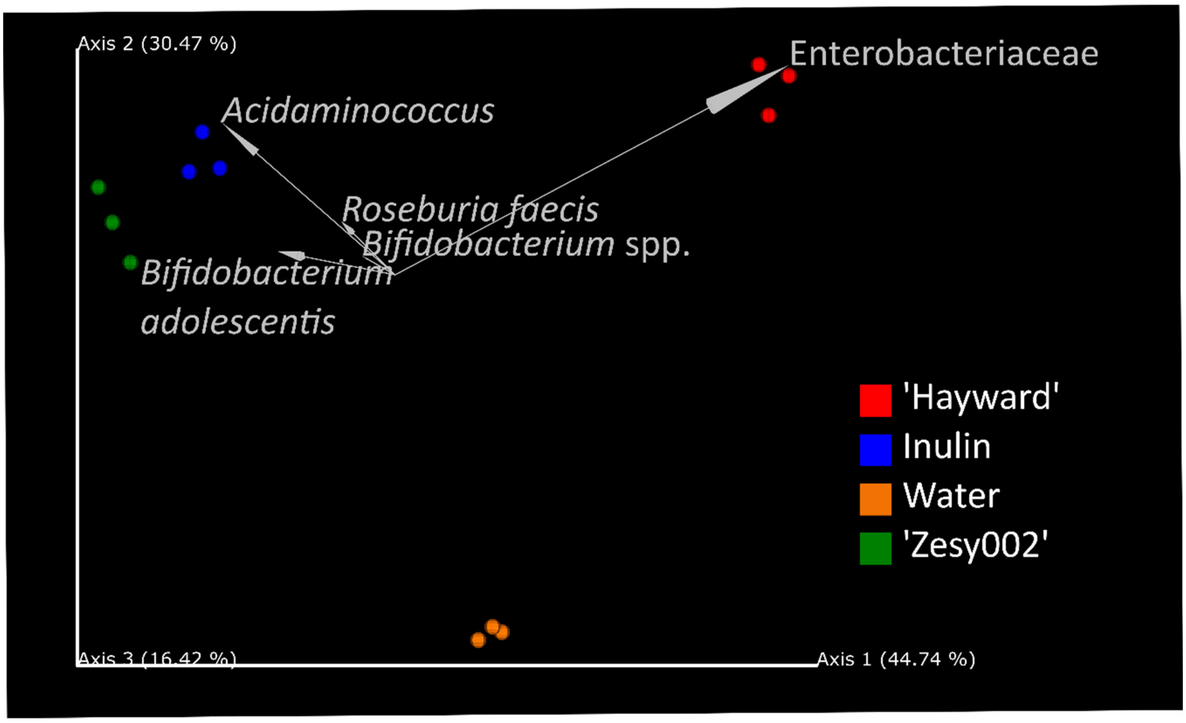

Figure S11. Principal co-ordinate analysis plot demonstrating β-diversity differences between samples, based on the Bray-Curtis index. The significance differentiating the effect of the treatments on changes in microbiome clusters was $p < 0.001$, PERMANOVA.

microorganisms

MDPI

Table S4. Relative changes in microbiome abundance in fermenta after 18 h of fermenta

		Log_2 fold change compared with water			Significance between substrates**			
Organism	*p*-value*	'Hayward'	'Zesy002'	Inulin	'Hayward'	'Zesy002'	Inulin	Water
Bifidobacterium spp.	3.27E-10	1.76	2.23	3.15	b	b	a	c
Bifidobacterium adolescentis	1.94E-65	1.43	3.21	0.65	b	a	c	d
Bifidobacterium longum	1.05E-43	1.80	1.89	0.43	a	a	b	c
Coriobacteriaceae	1.10E-07	1.85	1.77	1.19	a	a	b	c
Collinsella aerofaciens	7.45E-08	2.02	1.07	2.16	a	b	a	c
Barnesiellaceae	1.54E-03	-1.72	-0.53	-2.31	bc	ab	c	a
Butyricimonas spp.	4.57E-02	-5.00	-5.00	-3.54	ab	b	ab	a
Odoribacter spp.	4.93E-02	-0.93	-2.62	-0.83	ab	b	ab	a
Paraprevotellaceae	3.59E-02	-4.45	-4.35	-5.00	ab	ab	b	a
Bacteroides spp.	6.33E-18	-1.30	0.30	0.49	c	ab	a	b
Bacteroides caccae	1.77E-05	-1.77	-0.76	0.18	c	b	a	ab

Bacteroides fragilis	6.55E-07	-1.00	0.49	1.02	c	ab	a	b
Bacteroides ovatus	4.95E-10	-3.26	-1.41	-1.04	c	b	b	a
Bacteroides uniformis	1.62E-06	-1.10	-0.63	-0.37	c	b	ab	a
Parabacteroides spp.	3.35E-09	-2.46	-0.54	-0.85	c	ab	b	a
Prevotella copri	9.42E-31	4.34	-0.33	0.87	a	c	b	bc
Rikenellaceae	9.75E-03	-5.00	-4.82	-3.77	b	b	ab	a
Alistipes finegoldii	2.14E-02	-2.27	-1.73	-1.45	b	ab	ab	a
Alistipes onderdonkii	1.94E-20	-3.35	-3.37	-2.01	c	c	b	a
Enterococcus spp.	3.06E-03	-1.52	0.31	0.37	b	a	a	a
Mogibacteriaceae	5.13E-08	-1.59	-2.80	-2.81	b	b	b	a
Christensenellaceae	2.64E-03	-0.42	-0.47	-0.68	ab	b	b	a
Clostridiaceae	1.79E-03	2.64	-5.00	3.86	a	b	a	a
Clostridium spp.	2.16E-12	2.01	2.14	1.02	a	a	b	c
Ruminococcus spp.	1.76E-06	0.92	1.72	1.78	b	a	a	c

Ruminococcus gnavus	3.37E-08	0.59	-5.00	0.17	a	b	a	a
Lachnospiraceae	6.00E-05	-0.38	-2.53	-1.87	a	b	b	a
Blautia spp.	5.90E-07	-0.02	-0.84	0.70	b	c	a	b
Blautia obeum	4.82E-24	0.99	0.80	-0.45	a	a	c	b
Blautia producta	4.94E-02	1.78	1.02	1.53	b	ab	b	a
Clostridium colinum	1.35E-66	5.00	5.00	-0.75	a	b	c	c
Coprococcus spp.	5.43E-21	1.47	-3.77	1.50	a	c	a	b
Coprococcus catus	8.62E-26	-2.42	-4.06	-3.03	b	c	b	a
Coprococcus eutactus	9.48E-08	-0.39	-5.00	-4.65	a	b	b	a
Dorea spp.	5.85E-05	-0.80	-0.52	0.46	c	bc	a	ab
Dorea formicigenerans	4.16E-10	-1.22	-3.25	-1.97	b	c	b	a
Dorea longicatena	3.79E-21	0.01	-5.00	-2.87	a	c	b	a
Lachnospira spp.	7.05E-23	4.04	1.33	0.34	a	b	c	c
Roseburia	2.18E-04	-3.08	-2.21	-5.00	b	ab	c	a

Roseburia spp.	5.89E-17	-0.49	-5.00	-5.00	a	b	b	a
Roseburia faecis	1.04E-49	4.81	4.61	5.00	b	b	a	c
Ruminococcaceae	7.70E-04	-0.34	-1.56	-1.11	ab	c	bc	a
Ruminococcaceae (2)	1.07E-06	-0.57	2.82	-3.81	bc	a	c	b
Butyricicoccus pullicaecorum	2.57E-26	1.25	-0.97	0.02	a	c	b	b
Faecalibacterium prausnitzii	7.20E-18	2.09	0.49	0.20	a	b	b	b
Gemmiger formicilis	2.18E-10	0.81	0.87	0.95	a	a	a	b
Oscillospira spp.	5.86E-03	-3.08	-1.59	-1.95	b	ab	ab	a
Ruminococcus spp.	1.93E-04	-5.00	-0.66	-0.24	b	a	a	a
Ruminococcus bromii	3.86E-18	-0.94	0.18	0.49	c	b	a	b
Acidaminococcus spp.	2.53E-14	3.08	3.41	3.72	a	a	a	b
Dialister spp.	2.12E-08	2.26	1.18	1.25	a	b	b	c
Phascolarctobacterium spp.	5.78E-04	-1.39	-1.31	-0.91	b	b	b	a
Veillonella dispar	3.79E-07	-1.47	5.00	4.86	b	a	a	b

Erysipelotrichaceae	5.42E-04	0.17	-0.20	1.10	b	b	a	b
Sutterella spp.	1.50E-22	-1.26	-3.13	-3.31	b	c	c	a
Bilophila spp.	1.31E-10	-1.68	-1.24	-0.88	c	bc	b	a
Aeromonas spp.	5.17E-04	-1.60	-5.00	0.99	a	b	a	a
Enterobacteriaceae	1.87E-95	4.10	1.05	0.15	a	b	c	c
Serratia spp.	2.10E-03	1.33	-5.00	3.77	a	b	a	a
Pseudomonas spp.	2.34E-04	-1.08	-5.00	0.95	a	b	a	a
Akkermansia muciniphila	6.71E-04	-0.59	0.44	-0.02	c	a	b	b

Table S5. Mean counts of Caco-2 cell genes

Gene	Statistic	0 h (no fecal inoculum)	5 h	18 h	0 h (no fecal inoculum)	5 h	18 h	0 h (no fecal inoculum)	5 h	18 h	0 h (no fecal inoculum)	5 h	18 h	Fecal inoculum	Background media
		Water			Inulin			'Zesy002'			'Hayward'			Control	
ARNTL	Mean	105	134*	168*	58	74	129	109	133*	147*	51	61	56	41	41
	SE	23	22	20	28	27	41	20	20	34	28	18	26	17	20
CLDN4	Mean	4646	7971*	7039*	4979	3385	3762	5160	6141	6802*	4382	5678	6947*	2940	3294
	SE	1690	2514	2052	1230	247	603	2205	1310	2047	276	996	2050	222	298
ASMT	Mean	4	8	17*	4	2	3	5	8	4	3	7	6	1	3
	SE	2	3	13	2	1	1	1	3	2	1	3	1	0	1
IL10	Mean	4	10	23*	6	5	6	21*	9	8	6	9	10	4	3
	SE	2	3	11	5	3	1	19	4	2	0	6	5	2	1

MTNR1A	Mean	2	7	18	17	6	8	8	3	8	9	19	9	6	7
	SE	1	2	13	18	1	4	6	1	2	7	12	4	3	4
MTNR1B	Mean	3	5	18	8	1	3	11	3	3	4	5	10	2	3
	SE	1	2	14	7	0	0	8	2	1	1	2	7	1	1
PER1	Mean	31	21	43	52	31	55	39	24	23	37	23	26	20	29
	SE	6	4	21	24	5	5	17	3	5	7	8	4	2	5
TNF-α	Mean	17	15	27*	23*	14	17	34*	22*	18*	22*	15	16	9	7
	SE	2	0	7	14	4	1	25	6	3	3	3	4	5	3
CLDN1	Mean	1445	1762	1697	1346	985	1144	1366	2009	1990	966	1044	1116	966	1056
	SE	555	404	431	155	111	216	588	524	634	275	76	316	91	92
CLOCK	Mean	126	121	105	123	113	134	142	137	149	123	133	111	113	114
	SE	8	10	24	11	15	12	24	12	15	4	13	37	1	8
CRY1	Mean	282	213	314	263	278	360	292	252	294	281	200	225	233	211
	SE	71	41	74	105	54	57	116	48	48	44	34	43	100	76

CRY2	Mean	213	211	280	218	237	287	262	208	213	201	173	191	179	183
	SE	22	47	32	30	25	29	50	27	51	24	15	26	40	25
NOS2	Mean	534	566	393	429	370	414	449	595	556	443	414	282	389	340
	SE	47	122	120	18	44	11	35	124	93	63	10	68	105	46
OCLN	Mean	2068	2408	1824	2160	1571	1579	2218	2305	2457	1939	2275	1643	1616	1823
	SE	540	613	476	284	61	99	735	503	544	83	346	424	3	163
PER2	Mean	93	68	79	81	73	49	109	80	55*	86	58	56	68	76
	SE	10	10	27	30	5	6	1	17	9	16	6	7	15	22
PER3	Mean	354	307	275	321	234	288	338	366	320	257	206	206	199	200
	SE	48	14	33	83	87	90	74	18	35	60	70	80	68	86
SLC6A3	Mean	78	147*	108	60	109	114	93	155*	162*	75	101	114	75	65
	SE	23	16	25	6	21	19	18	27	22	22	26	29	6	13
TPH1	Mean	24	32	197*	64	16	32	68	30	43	40	43	63	18	21
	SE	8	3	188	57	3	3	43	8	18	15	15	20	2	3

TPH2	Mean	20	17	27	31	5	9	67*	19	16	29	18	22	13	12
	SE	1	6	8	13	2	5	35	10	8	18	9	9	1	5

[1]Mean counts ≤ 5 are in the range of negative probe controls.
[2]Pairwise adjusted *p*-values were calculated by contrasts in DESeq2, and adjusted for false discovery rate within each gene (size factors were set to 1 for all samples).
[3]Significance difference ($p < 0.05$) from Background Media Control is indicated by *.
[4]There were no significant differences between substrates at each fermenta time point.
[5]Significance difference between fermenta within a substrate group were $p = 0.034$ for *TPH2* with 'Zesy002' fermenta and $p = 0.002$ for *TPH1* with water.

1. Waller, D.G.; Sampson, A.P. Extrapyramidal movement disorders and spasticity. In *Medical Pharmacology and Therapeutics (Fifth Edition)*, Waller, D.G.; Sampson, A.P., Eds. Elsevier: 2018; pp 325-336.
2. Baj, A.; Moro, E.; Bistoletti, M.; Orlandi, V.; Crema, F.; Giaroni, C. Glutamatergic signaling along the microbiota-gut-brain axis. *Int. J. Mol. Sci.* **2019**, *20*, 1482.
3. Boonstra, E.; de Kleijn, R.; Colzato, L.S.; Alkemade, A.; Forstmann, B.U.; Nieuwenhuis, S. Neurotransmitters as food supplements: the effects of GABA on brain and behavior. *Front. Psychol.* **2015**, *6*, 1520-1520.
4. Gnegy, M.E. Chapter 14 - Catecholamines. In *Basic Neurochemistry (Eighth Edition)*, Brady, S.T.; Siegel, G.J.; Albers, R.W.; Price, D.L., Eds. Academic Press: New York, 2012; pp 283-299.
5. Kozich, J.J.; Westcott, S.L.; Baxter, N.T.; Highlander, S.K.; Schloss, P.D. Development of a dual-index sequencing strategy and curation pipeline for analyzing amplicon sequence data on the MiSeq Illumina sequencing platform. *Appl. Environ. Microbiol.* **2013**, *79*, 5112.
6. Bolyen, E.; Rideout, J.R.; Dillon, M.R.; Bokulich, N.A.; Abnet, C.C.; Al-Ghalith, G.A.; Alexander, H.; Alm, E.J.; Arumugam, M.; Asnicar, F., *et al.* Reproducible, interactive, scalable and extensible microbiome data science using QIIME 2. *Nat. Biotechnol.* **2019**, *37*, 852-857.
7. Callahan, B.J.; McMurdie, P.J.; Rosen, M.J.; Han, A.W.; Johnson, A.J.A.; Holmes, S.P. DADA2: High resolution sample inference from Illumina amplicon data. *Nat. Methods* **2016**, *13*, 581-583.
8. Janssen, S.; McDonald, D.; Gonzalez, A.; Navas-Molina, J.A.; Jiang, L.; Xu, Z.Z.; Winker, K.; Kado, D.M.; Orwoll, E.; Manary, M., *et al.* Phylogenetic placement of exact amplicon sequences improves associations with clinical information. *mSystems* **2018**, *3*, e00021-00018.
9. DeSantis, T.Z.; Hugenholtz, P.; Larsen, N.; Rojas, M.; Brodie, E.L.; Keller, K. Greengenes, a chimera-checked 16S rRNA gene database and workbench compatible with ARB. *Appl Environ Microbiol* **2006**, *72*.
10. Kaeffer, B.; Pardini, L. Clock genes of mammalian cells: Practical implications in tissue culture. *In Vitro Cell. Dev. Biol. anim.* **2005**, *41*, 311-320.
11. Love, M.I.; Huber, W.; Anders, S. Moderated estimation of fold change and dispersion for RNA-seq data with DESeq2. *Genome Biol* **2014**, *15*, 550.

by the authors. Licensee MDPI, Basel, Switzerland. This article is an open access article distributed under the terms and conditions of the Creative Commons Attribution (CC BY) license (http://creativecommons.org/licenses/by/4.0/).
